# Stable individual characteristics in the perception of multiple embedded patterns in multistable auditory stimuli

**DOI:** 10.3389/fnins.2014.00025

**Published:** 2014-02-28

**Authors:** Susan Denham, Tamás M. Bõhm, Alexandra Bendixen, Orsolya Szalárdy, Zsuzsanna Kocsis, Robert Mill, István Winkler

**Affiliations:** ^1^Cognition Institute, University of PlymouthPlymouth, UK; ^2^School of Psychology, University of PlymouthPlymouth, UK; ^3^Research Centre for Natural Sciences, Institute of Cognitive Neuroscience and Psychology, Hungarian Academy of SciencesBudapest, Hungary; ^4^Department of Telecommunications and Media Informatics, Budapest University of Technology and EconomicsBudapest, Hungary; ^5^Auditory Psychophysiology Lab, Department of Psychology, Cluster of Excellence “Hearing4all”, European Medical School, Carl von Ossietzky University of OldenburgOldenburg, Germany; ^6^Department of Cognitive Science, Budapest University of Technology and EconomicsBudapest, Hungary; ^7^Institute of Psychology, University of SzegedSzeged, Hungary

**Keywords:** auditory scene analysis, multistability, auditory streaming, perceptual switching, individual differences

## Abstract

The ability of the auditory system to parse complex scenes into component objects in order to extract information from the environment is very robust, yet the processing principles underlying this ability are still not well understood. This study was designed to investigate the proposal that the auditory system constructs multiple interpretations of the acoustic scene in parallel, based on the finding that when listening to a long repetitive sequence listeners report switching between different perceptual organizations. Using the “ABA-” auditory streaming paradigm we trained listeners until they could reliably recognize all possible embedded patterns of length four which could in principle be extracted from the sequence, and in a series of test sessions investigated their spontaneous reports of those patterns. With the training allowing them to identify and mark a wider variety of possible patterns, participants spontaneously reported many more patterns than the ones traditionally assumed (Integrated vs. Segregated). Despite receiving consistent training and despite the apparent randomness of perceptual switching, we found individual switching patterns were idiosyncratic; i.e., the perceptual switching patterns of each participant were more similar to their own switching patterns in different sessions than to those of other participants. These individual differences were found to be preserved even between test sessions held a year after the initial experiment. Our results support the idea that the auditory system attempts to extract an exhaustive set of embedded patterns which can be used to generate expectations of future events and which by competing for dominance give rise to (changing) perceptual awareness, with the characteristics of pattern discovery and perceptual competition having a strong idiosyncratic component. Perceptual multistability thus provides a means for characterizing both general mechanisms and individual differences in human perception.

## Introduction

Most sound sources of interest in the world around us emit sequences of sound events, e.g., the notes in a birdsong or the words spoken in conversation. These sounds are seldom present in isolation and typical sound events consist of many time-varying components. The problem for auditory perception is to parse this complex scene, and to do so in a timely manner that allows us to interact appropriately with the sound emitting objects of interest. The problem of grouping, both the simultaneously present components that belong to the same sound event, and the sequential associations between events emitted by the same source, is known as auditory scene analysis (Bregman, 1990). Understanding this seemingly effortless process of perceptual organization is an essential step toward explaining what determines our conscious perceptions of the world. Most of the studies in this field to date have focused on trying to identify the general processing strategies used by the human brain for parsing the auditory scene. In doing so, inter-individual differences have typically been treated as a source of noise in the experimental data, as is the case in many cognitive studies (cf. Kanai and Rees, 2011). In the current study, we asked whether the patterns of responses obtained from individual listeners are stable characteristics of the person.

Sequential grouping in auditory scene analysis has typically been studied using the auditory streaming paradigm: ABA-ABA-, where A and B denote different sounds and “-” stands for a silent interval with the same duration as the two sounds (van Noorden, 1975; Bregman, 1990). There has been a long-standing assumption that in listening to such a sound sequence, listeners make a perceptual decision between *integration* (the grouping of all sounds into the same stream, perceived as a repeating ABA- pattern) and *segregation* (the parsing of the sequence into two separate streams, perceived as repeating A- and B--- patterns, with one in the foreground and the other in the background). This perceptual decision is influenced by the distinctiveness (or similarity) of the A and B tones (e.g., differences in frequency, location or timbre) and the rate at which the sounds are presented (for a review see Moore and Gockel, 2012). The trade-off between similarity and presentation rate has led to the suggestion that in audition the Gestalt principle of similarity (Köhler, 1947) is mediated by time; i.e., similarity and good continuation combine to determine the likelihood of sequential grouping (Jones, 1976; Winkler et al., 2012; Denham and Winkler, 2014).

In most studies it has been assumed that *integration* and *segregation* are the only perceptual organizations possible, and that they are mutually exclusive (van Noorden, 1975). In addition, because it was thought that integration was always perceived first and that the build-up of segregation was a rather slow process (on the order of several seconds) (Anstis and Saida, 1985), it was suggested that perceptual organization could be viewed as a process in which the auditory system accumulates evidence in favor of an appropriate perceptual decision (Bregman, 1990). However, a number of recent experiments have challenged these ideas. Firstly, it has been found that there are other possible perceptual organizations, and, when given the possibility to do so, listeners also report hearing repeating patterns, sometimes for rather long periods of time, which do not match either of the patterns described above (Bendixen et al., 2010; Denham et al., 2013). Secondly, rather than fixing on a single perceptual decision, given sufficient time, perception switches between alternative interpretations of the sequence (Denham and Winkler, 2006; Pressnitzer and Hupé, 2006) and does so for all combinations of frequency difference and presentation rate tested to date, even those that have been assumed to be strongly biased toward either *integration* or *segregation* (Denham et al., 2013). Thirdly, *segregation* is often reported first for some parameter combinations (Deike et al., 2012; Denham et al., 2013), and the gradual build-up of *segregation* has been shown to be to some extent an artifact of the analysis and visualization methods used (Deike et al., 2012).

Based on these new findings it has been proposed that perceptual organization is a process in which the auditory system continually attempts to discover patterns (or regularities) in the incoming sequence (Winkler et al., 2012; Denham and Winkler, 2014). Multiple such patterns may be detected embedded in a sequence and represented in parallel. Consistent with theories of binocular rivalry (for a review see Logothetis et al., 1996), the proposal is that a sequence of conscious perceptual states arises as a result of ongoing competition for perceptual dominance between concurrent rivaling percepts; evidence for which has been found in an auditory mismatch negativity experiment (Horváth et al., 2001). The ease with which each pattern is discovered (related to the notion of similarity described above) determines how likely it is that the pattern will be perceived, especially at the beginning of a sound sequence. The auditory system uses each detected pattern to generate expectations of future events, that, if violated, signal new (i.e., as yet unmodeled) information in the sequence. Patterns that predict the same events compete for dominance. Compatible patterns, i.e., those that do not attempt to predict the same events, form cooperative groups that give rise to the perceptual organizations reported by listeners. Perceptual switching between these cooperative groups, and the corresponding changes in perceptual awareness, are caused by the competition between incompatible patterns (Winkler et al., 2012). A computational model based on these principles successfully replicated many of the phenomena of perceptual bi-stability in the auditory streaming paradigm (Mill et al., 2013).

## Experiment 1

In our previous experiments, having realized that participants sometimes experienced organizations other than *integration* and *segregation*, we used instructions that provided participants with a wider range of possible reports (e.g., see Bendixen et al., 2010; Denham et al., 2013). Participants were instructed to report *integration* if all tones in the sequence were perceived as belonging to a single repeating pattern (i.e., a single stream); *segregation*, if the sequence was perceived as consisting of two repeating patterns (or streams), one containing only the A tones and the other only the B tones; *both*, if the sequence was perceived as consisting of two streams, one containing both A and B tones, and the other only A or only B tones; and finally, *none*, if no repeating pattern was detected. Over the course of many experiments we found that (1) *both* is reported on average between 10 and 30% of the total duration, (2) *both* is almost never reported as the first percept, (3) the incidence of *both* percepts varies considerably between listeners, and (4) there is a tendency for *both* reports to be more common for parameter combinations supporting more evenly balanced proportions of *integration* and *segregation* (Bendixen et al., 2010, 2013; Denham et al., 2013; Szalárdy et al., 2013).

**Figure 1:**
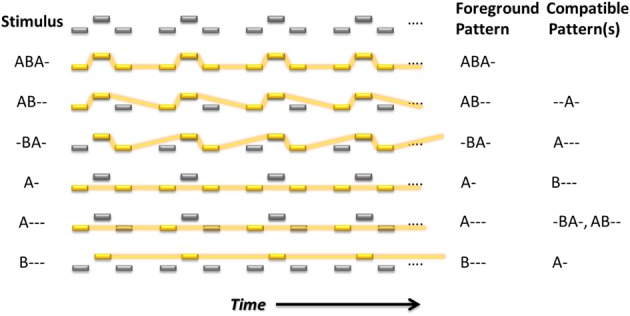
**Embedded repeating patterns up to length four that can, in theory, be perceived by listeners in the auditory streaming paradigm**. Yellow rectangles indicate the foreground pattern. Feature differences, in this case frequency differences, are indicated by displacement in a vertical direction for the tones in each sequence. The foreground and compatible (background) patterns for each dominant (emphasized) pattern are indicated to the right in letter form.

Although these experiments have supported the idea that perceptual organizations other than *integration* and *segregation* can be perceived when listening to the ABA- sequence, precisely what patterns listeners perceive when they report *both* has not been previously investigated. One possibility is that only *integration* and *segregation* are perceived but there is sometimes very rapid switching between them, and since there is some sluggishness in the system, listeners simply report *both*. In this case we would expect to find no explicit reports of distinctive patterns other than ABA-, A-, or B---. Another possibility, suggested by our modeling studies, is that the auditory system finds many patterns embedded even within this simple sequence, which results in the emergence of other compatible groups and thus other perceptual organizations; see Figure 1. In the experiments reported here, we investigated whether listeners spontaneously report perceiving these more uncommon patterns and, if so, to what extent their perception is influenced by stimulus parameters.

In order for participants to be able to quickly and reliably report what they perceived and to associate the possible patterns with the user interface controls, each participant attended a series of training sessions prior to the commencement of the main experiment. Previous work (Rogers and Bregman, 1993; Snyder et al., 2008, 2009b; Haywood and Roberts, 2011, 2013) has shown strong contextual effects of prior learning on auditory streaming. However, these studies of contextual influences on perception have typically involved within-session manipulations, and the perceptual effects have been probed using stimuli of rather short duration (generally <10 s). To our knowledge the experiments in this report are the first to include extensive multi-session training. In addition, we are concerned here not with differential effects of prior training on perception, but rather with the reliability of participants' ability to correctly categorize the specific patterns that may occupy their perceptual awareness.

Although the characteristics of perceptual switching are known to be very stochastic (Levelt, 1968), it is also known that some characteristics, e.g., typical switching rates, may be rather idiosyncratic (Aafjes et al., 1966; Kanai et al., 2010). We therefore investigated whether there was internal consistency in the data; i.e., whether individual listeners' perceptual switching behavior was similar across sessions. If so, this would provide some measure of confidence that listeners were reporting what they perceived, and were reliably engaged in the task. Addressing this question required us to conduct numerous (10 or more) sessions with each individual listener, and hence we involved only a small number of experimental participants (*N* = 6).

In summary, the two aims of the study were: (1) to determine whether listeners at least occasionally experience sequences created according to the auditory streaming paradigm in terms of percepts outside the traditional *integrated* and *segregated* sound organizations, and (2) to assess whether the perceptual reports of individual listeners show stable consistent characteristics (i.e., whether the patterns of perceptual reports are more similar within a single listener across sessions than between different listeners).

### Methods

#### Participants

Six healthy volunteers (mean age 22.3 years, range 19–25 years; all right-handed; 4 male, 2 female) took part in Experiment 1, which was conducted over numerous sessions over a period of approximately 1 month. All participants had reportedly normal hearing. None of the participants were taking any medication affecting the central nervous system. In compliance with the Declaration of Helsinki, participants gave written informed consent after the experimental procedures had been explained to them. Participants received modest financial compensation for their participation.

#### Stimuli

Sinusoidal tones of 75 ms (ms) duration (including 10 ms rise and fall times) and with an intensity of 40 dB sensation level (above hearing threshold, adjusted individually for each participant) were arranged according to the auditory streaming paradigm (a cyclically repeating “ABA-” pattern) in five stimulus conditions, with frequency difference (Δ*f*) and stimulus onset asynchrony (SOA, onset to onset time interval) as follows: (1) Δ*f* = 3 semitones (st), SOA = 100 ms; (2) Δ*f* = 16 st, SOA = 100 ms; (3) Δ*f* = 7 st, SOA = 150 ms; (4) Δ*f* = 3 st, SOA = 200 ms; (5) Δ*f* = 16 st, SOA = 200 ms. The frequency of the “A” tones was 400 Hz, and the frequency of the “B” tones was *n* semitones higher, depending on condition. At each test session, participants were presented with 10 min long ABA- tone sequences, one for each of the five conditions, delivered in a randomized order. An extra 30 s verification segment (see the *Test procedure* section) was appended to the end of each 10-min long stimulus block.

#### Apparatus and procedures

**Figure 2:**
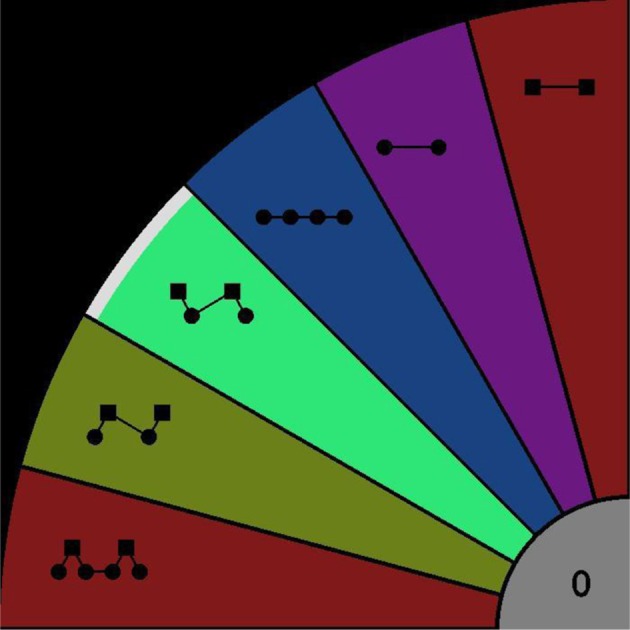
**Response interface**. Participants were instructed to press the colored area corresponding to their dominant (foreground) percept, as indicated by the icons. Starting from the lowest area these patterns can be expressed using letters as ABA-, AB--, -BA-, A-, A---, B---, *confused* (gray area “0”). In this screen shot, the -BA- pattern is being reported as the currently perceived pattern (indicated by higher intensity and the white segment at the outer edge of the region).

Participants were seated in an acoustically shielded chamber. Sounds were presented binaurally via headphones. Responses were given using a touch-screen monitor. As illustrated in Figure 1, restricting the patterns to length no longer than four, there are six possible patterns that can be extracted from the ABA- sequence; ABA-, AB--, -BA-, A-, A---, and B---. A specific area of the display was assigned to each of these response options (indicated by color and graphical icon). A further area (gray, “0”) allowed listeners to indicate when they could not decide between the patterns (*confused*). Participants were required to use the index finger of their right hand to press the button corresponding to the pattern they experienced, and to keep the button depressed for as long as they continued hearing the pattern. The interface did not allow multiple responses to be reported simultaneously. A screenshot of the response screen with one “response button” pressed can be seen in Figure 2.

#### Training procedure

In order to make sure that listeners understood how each pattern sounds, and could reliably report their percepts, they attended a number of training sessions before the main experiment. In these sessions they learnt to use the response interface, and to report the dominant (foreground) pattern that they perceived. A detailed log of each participant's training history was kept. The experimenter screened performance to decide when to stop training. Only once listeners could reliably report the entire set of patterns were they ready to take part in the main experiment.

In each training session the experimenter adaptively adjusted the training procedure in accord with the participants' level of understanding and performance. Two pattern repetition speeds were pre-assigned by varying the silent period; slow ABA-- and normal ABA-, where “-” is a silent period corresponding to the SOA. Participants started with putatively easier tasks, and then proceeded to more difficult ones as their performance improved. The teaching procedure started with a demonstration in which the participants listened to each of the possible patterns where the tones not included in the pattern were left out and the corresponding button was depressed on the screen. Next, they were presented with blocks in which the order of the patterns were randomized and the duration of the patterns were also different. Participants had to press the button corresponding to their percept. These blocks were repeated for as long as the experimenter considered it necessary.

Next, the different patterns were introduced by means of emphasis, i.e., tones not part of the pattern were attenuated (−18 dB). Once again participants were asked to observe each of the possible pattern and button combinations, as explained above. Then they were presented with blocks in which the order of the patterns were randomized and the duration of the patterns were also different. Participants had to press the button corresponding to their percept. These blocks were again repeated for as long as the experimenter considered it necessary.

Over the course of three or four sessions these tasks were repeated until the participants reached the point where they were able to confidently identify the patterns. Finally, one or two blocks identical to the test procedure were administered to prepare them for the test sessions.

#### Test procedure

Once their training had been completed, participants attended seven test sessions spread over a period of approximately 1 month with at least 2 days between consecutive sessions.

At the start of each test session the experimenter made sure that participants remembered the patterns they were required to report using both auditory and visual illustrations. They were also reminded of their task by means of two to four training blocks in which the various patterns were emphasized, as described above.

Participants were instructed to listen to the tone sequences and to continuously indicate their percepts by depressing the region of the touch-screen corresponding to the pattern that was currently most prominent. Participants were encouraged to employ a neutral listening set, and to refrain from attempting to hear out one or another pattern. A break of at least 30 s separated successive stimulus blocks, with additional time given to participants as needed. Each test session lasted up to 1.5 h.

A criticism that has been leveled at the auditory streaming paradigm is that it relies on listeners being able to make accurate subjective reports of their perceptions. It is of course difficult to verify whether someone is actually reporting what they hear, rather than simply pressing buttons randomly or in order to satisfy the experimenter. We tried to address this issue in two ways. Firstly, an extra 30 s verification segment was appended to the end of each 10-min long stimulus block. In these verification segments, one of the six patterns (ABA-, AB--, -BA-, A-, A---, B---), randomly chosen, was emphasized by attenuating all non-pattern tones (−18 dB); i.e., the stimulation was identical to those used for training. If listeners are correctly reporting their percepts, then they should report the emphasized pattern during this period. Secondly, listeners performed the main experiment repeatedly and on different occasions (i.e., in seven separate sessions spread over a period of approximately 1 month). The rationale was that random button pressing should not lead to consistent response patterns across sessions. Altogether, including training sessions, four participants took part in 10 sessions, while two participants took part in 11.

#### Data recording and analysis

The state of the response buttons was continuously recorded at a nominal sampling rate of 250 Hz. However, due to the use of the graphical user interface for collecting data, it was not possible to guarantee a strictly regular sampling. For this reason the raw data was resampled at a regular 4 ms sampling period. Before analysing the button presses, because there was an explicit button for participants to use if they were confused or heard none of the patterns, we removed the phases where no button was pressed. In addition, all cases in which the duration between successive changes in response (termed a *perceptual phase*) was shorter than 300 ms were discarded because these were assumed not to result from intentional reports (Moreno-Bote et al., 2010). In Experiment 1 the data removed in these ways amounted to 3.6% of the total data duration.

To check that participants were performing the task correctly, the verification data was analyzed to determine the total proportion of time spent reporting the emphasized pattern. The latency for switching to the emphasized pattern from the start of the verification section was also extracted in order to take account of the time taken for the emphasized pattern to overcome the dominance of whatever pattern participants perceived at the time the verification section started. The combination of these two durations gives a good account of the accuracy with which participants could identify and report each of the patterns.

To investigate the dynamics of the discovery of alternative patterns, the latency for the first report of each pattern was extracted for each participant for each session and condition. It is not necessarily the case that all patterns are experienced, but if they are this gives a measure of how quickly they are discovered by the auditory system. Perceptual phase durations in both auditory and visual multistability experiments are typically log normally distributed (Pressnitzer and Hupé, 2006). Therefore, mean phase durations are usually calculated by finding the mean value in the log domain and then converting back to the linear domain. However, here we found that in some cases the duration data (especially the latency to the first report of each pattern) was not log normally distributed, so for consistency, summary durations are given as the median of the corresponding data.

To characterize the perceptual switching patterns of participants in response to the tone sequences and to analyse the parameter dependence of perception, transition matrices were constructed using the method described by Denham et al. (2012). Each transition matrix is a 7 × 7 matrix with elements that represent the probability of switching from one percept to another percept (i.e., seven possibilities in all: six patterns and *confused*), with the percept of origin corresponding to the column and the destination percept to the row of the element. A *global transition matrix* that summarizes all of the switching patterns found in the experiment was constructed by counting the number of occurrences of each transition for all of the data recorded during the experiment. From this matrix, the overall proportions and phase durations of each percept were extracted. A set of five *condition transition matrices* were constructed by counting the number of occurrences of each possible transition, pooling all participants and all test sessions for each condition separately. To analyse individual differences between participants, *participant transition matrices* were constructed, one per participant per session. The participant transition matrices were calculated by counting the number of occurrences of each possible transition, pooling responses from all conditions for each participant within each test session. As explained in Denham et al. (2012), the global transition matrix provides neutral default values in the case of missing transitions for the more restricted data sets used to construct the condition and participant matrices.

Individual differences were investigated using the participant transition matrices. The Kullback-Leibler divergence (Kullback, 1959) between each matrix from an individual participant (intra-participant distances) and between each individual participant's matrices and all other participants' transition matrices (inter-participant distances) was used as a measure of similarity between perceptual switching patterns. This is a richer characterization of perceptual switching than the switching rate measure usually used in this regard.

The overall distributions of the proportions of each of the patterns were compared using a repeated-measures ANOVA. The effects of condition on the proportion of each pattern were analyzed using a repeated-measures ANOVA followed by *post-hoc* pairwise comparisons using a pairwise sign test to analyse the influence of stimulus parameters on the proportion of *segregated*, *integrated* and *both* phases. The distributions of overall phase durations were analyzed using a repeated-measures ANOVA and pairwise Wilcoxon rank sum tests. Wilcoxon rank sum tests were used to compare cumulative latency distributions. Finally, the distributions of intra- vs. inter-individual differences were compared separately for each participant using a Wilcoxon rank sum test. The significance of all statistical tests was assessed at the 95% confidence level (α = 0.05). All analyses were carried out using Matlab and the Matlab Statistics Toolbox.

### Results

#### Unusable data

Three sessions were affected by problems with the user interface, as indicated by the occurrence of blocks in which no responses were recorded. These sessions (participant 1, session 8, participant 2, sessions 7 and 8) were excluded from the analysis.

#### Verification data

**Figure 3:**
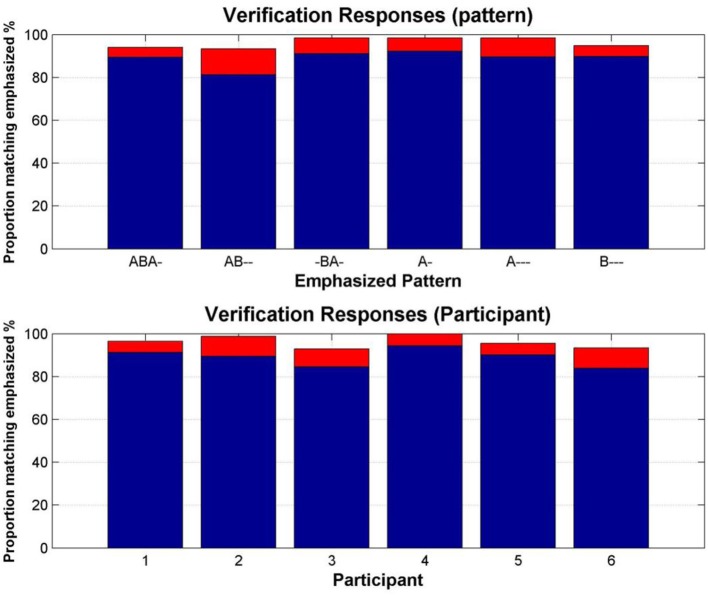
**Verification of pattern recognition. Top:** Proportion of the verification sections in which each emphasized pattern was reported. **Bottom:** Proportion of the verification sections in which each participant reported the emphasized patterns. Blue bars show the mean proportion of matching responses, red bars show the additional proportion accounted for by the latency to the first switch to the matching response.

The mean proportion of the total duration of the verification responses (i.e., those made during the 30 s verification sequences appended to the end of the test block, in which particular patterns were emphasized) matching the emphasized patterns was 88.9%; 96.2%, if the latency to switch to the emphasized pattern is included. The latency to the first switch to the emphasized response accounted for a mean of 2.2 s. Figure 3 shows this data according to pattern and according to participant. Overall, these “catch” sections showed that participants reliably categorized each emphasized pattern.

#### Proportion of each perceptual pattern

**Figure 4:**
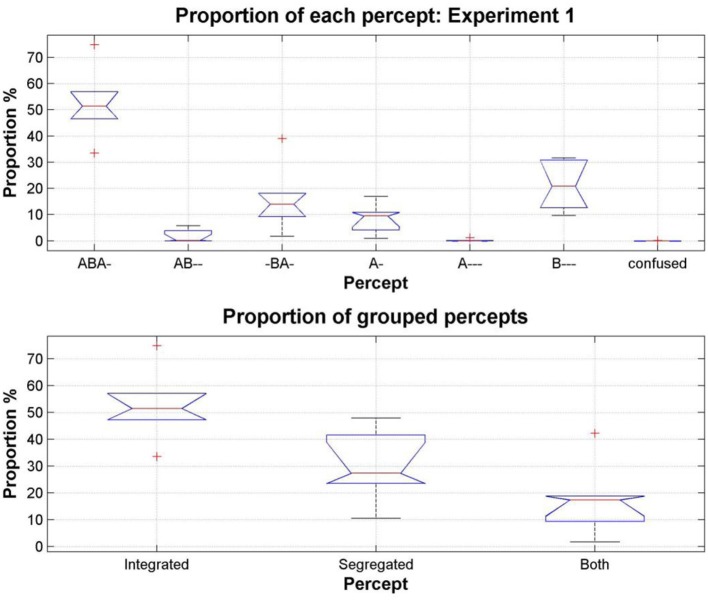
**Total proportion of each pattern, pooling the data from all participants, all sessions and all conditions in Experiment 1**. On each box, the central red line is the median, the upper and lower edges of the boxes are the 25th and 75th percentiles, the whiskers extend to the most extreme data points considered not to be outliers; outliers are plotted individually as red crosses. **Top:** Proportion of each pattern reported by participants (median values: ABA- 51.39%, AB-- 0.13%, -BA- 14.02%, A- 9.49%, A--- 0.02%, B--- 20.78%, *confused* 0.0008%). **Bottom:** Patterns grouped according to the perceptual organizations that participants were allowed to report in our previous studies: *integrated* (ABA-), *segregated* (A-, B---), and *both* (AB--, -BA-, A---).

The first question we wished to address was whether participants would perceive all of the patterns they encountered during training, or whether only the conventional patterns of *integration* (ABA-) and *segregation* (A-, B---) would be reported. The perceptual reports of all participants, across all test sessions and all conditions were pooled to investigate the overall occurrence of each pattern. We found that all of the patterns in the training data were reported during the experimental sequences. The distribution of the proportions of each varied widely [comparing the pattern proportions using a One-Way repeated-measures ANOVA, *F*_(6, 35)_ = 30.85, *p* < 0.0001]; Figure 4 (top). Pairwise comparisons between patterns show that all pattern proportions (except AB-- and A--- *p* = 0.2, AB-- and *confused p* = 0.14, -BA- and A- *p* = 0.22, -BA- and B--- *p* = 0.46, and A--- and *confused*, *p* = 0.32) are significantly different from each other, *p* < 0.05. Figure 4 (bottom), shows the proportions resulting from pooling the response alternatives into the categories used in our previous studies, *integrated*, *segregated* and *both (none* was excluded because of the very low incidence of *confused*, and the removal of all instances of *no button press)*; these proportions are comparable with those found in previous experiments (e.g., Bendixen et al., 2010; Denham et al., 2013).

**Figure 5:**
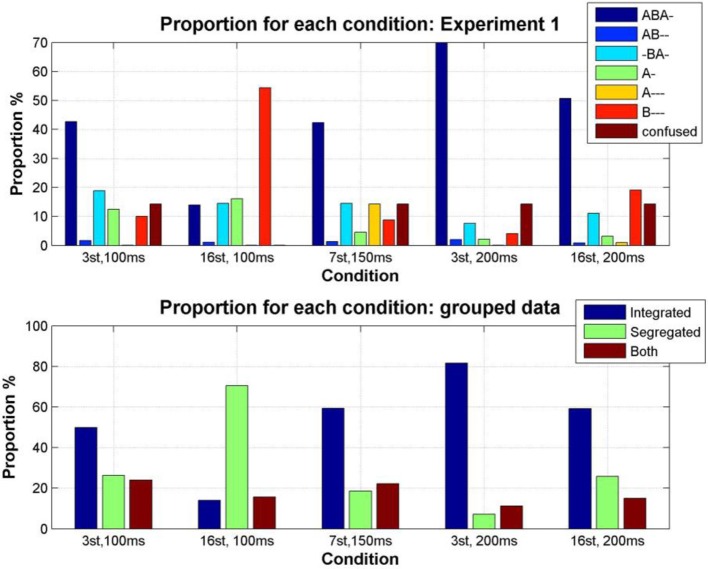
**Proportion of the total stimulus duration during which each pattern was perceived (color coded as indicated by the accompanying legend) for each condition, calculated from the condition transition matrices constructed by pooling the data from all participants for each condition (Denham et al., 2012)**. **Top:** Proportion of each pattern reported by participants. **Bottom:** Proportion of each pattern grouped according to the perceptual organizations that participants were allowed to report in our previous studies; *integrated* (ABA-), *segregated* (A-, B---), and *both* (AB--, -BA-, A---).

The parameter dependence of the proportion of time during which each pattern was perceived was analyzed using the condition transition matrices, both for the original responses and for the pooled responses (i.e., pooled for the response categories used in previous studies). As expected from previous experiments (e.g., Denham et al., 2013), a fast rate of change of stimulus parameters (Δ*f* = 16 st, SOA = 100 ms) increases the proportion of *segregation* [comparing the effect of condition using a One-Way repeated-measures ANOVA, *F*_(4, 25)_ = 12.7, *p* < 0.0001; pairwise sign test comparing the proportion of *segregation* in condition 2 vs. the proportion in every other condition, all *p* < 0.05], a slow rate of change of stimulus parameters (Δ*f* = 3 st, SOA = 200 ms) increases the proportion of *integration* [comparing the effect of condition using a One-Way repeated-measures ANOVA, *F*_(4, 25)_ = 13.96, *p* < 0.0001; pairwise sign test comparing the proportion of *integration* in condition 4 vs. proportion in every other condition, all *p* < 0.05]. By considering the grouped percepts, we can also see that if the patterns hypothesized to correspond to the *both* responses in previous experiments are pooled, then we find that there tends to be a higher incidence of *both* in the regions of intermediate rate of feature change, here conditions 1, 3, and 5. This seems to confirm Denham et al's., (2013) observations; however, a One-Way repeated-measures ANOVA did not show a significant effect of condition, *F*_(4, 25)_ = 0.73, *p* > 0.5, therefore the planned pairwise comparisons were not performed.

#### Phase duration of each perceptual pattern

**Figure 6:**
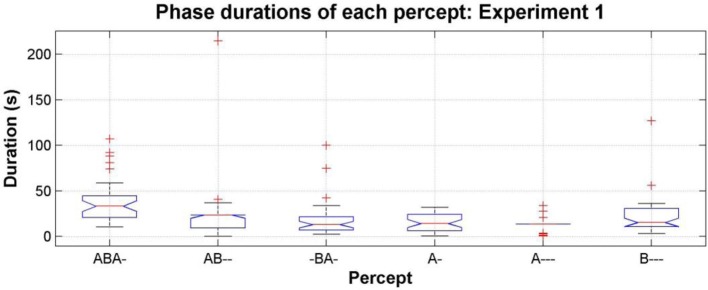
**Phase durations of each pattern, pooling the data from all participants, all sessions and all conditions in experiment 1; see Figure 4 for figure conventions**.

Another way to explore the data is to consider the statistics of perceptual phase durations. Phase durations can provide insights into the dynamics of perceptual switching not always apparent from the proportions; the same proportions can be achieved by many short phases or by fewer longer phases. In Figure 6 the median phase durations for each pattern are shown for the entire data set; for clarity the *confused* phases are omitted from this analysis. This plot shows that although the proportion of the patterns (AB--, -BA-, and A---) is rather low, if participants do report them, then the phase durations during which they are experienced can be comparable with those of the *segregated* percepts [comparing the median phase durations of the six different patterns using a One-Way repeated-measures ANOVA, *F*_(5, 30)_ = 2.32, *p* = 0.06; pairwise Wilcoxon rank sum test comparing distributions shows the durations of AB-- and -BA- not to be not significantly different from any other pattern, *p* > 0.05 for all comparisons, and the durations of A--- not to be significantly different from any other pattern, *p* > 0.05 for all comparisons except ABA-, *p* = 0.004].

#### Latency

**Figure 7:**
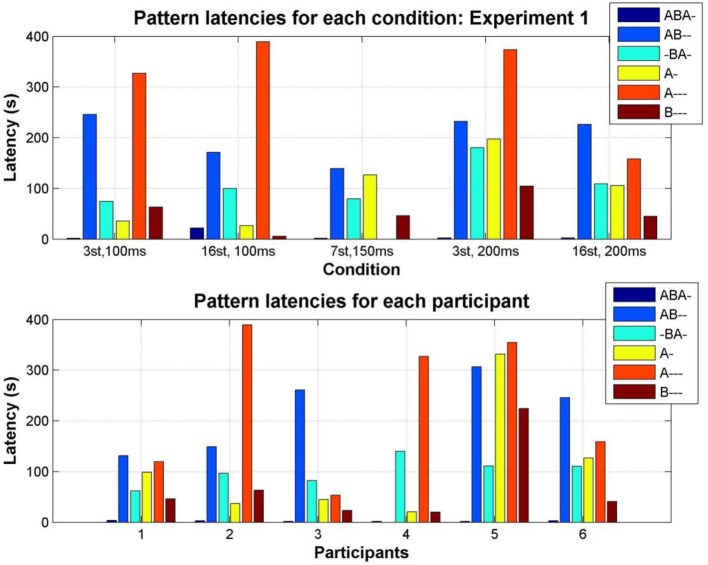
**Median latency of each pattern (color coded as indicated by the accompanying legend)**. **Top:** Median latency for the first report of each pattern in each condition, pooling all participants. **Bottom:** Median latency for the first report of each pattern by each participant, pooling all conditions.

The time taken to discover each of the perceptual patterns varied as a function of stimulus parameters (condition) and between individual participants; see Figure 7. With the exception of the A--- pattern, all patterns are eventually reported for all conditions, and all participants (with the exception of participant 4: AB--) reported all of the patterns at some time during the experiment.

**Figure 8:**
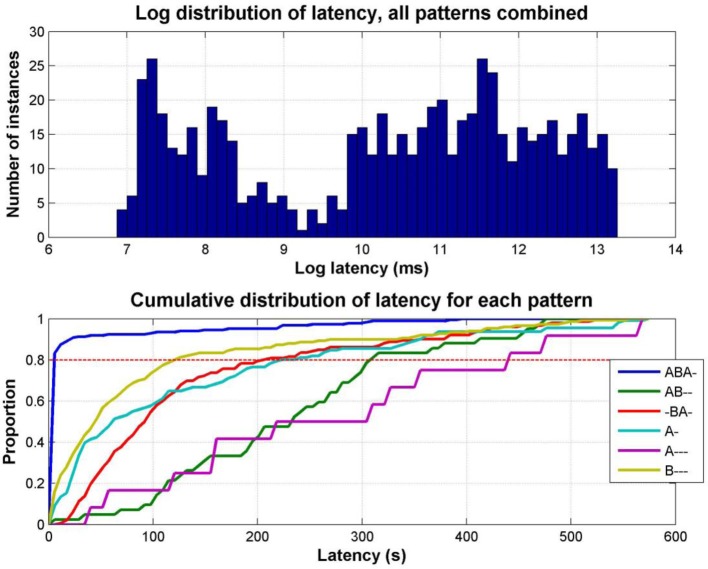
**Distribution of response latencies**. **Top:** Distribution of latencies for all patterns combined, for all data in Experiment 1; for clarity latency is plotted as the log of the latency in milliseconds. **Bottom:** Cumulative distribution of latency for each pattern separately, combining data from all participants and sessions. Each line represents the proportion of latencies of the pattern indicated by the color code (see legend) less than the latency indicated along the x axis. For example, at a latency of 100 s, less than 20% of first reports of A--- have occurred, while more than 90% of first ABA- reports have occurred.

The latencies for reporting each pattern are not normally distributed; plotting the latency distribution in the log domain clearly shows its bimodal nature (Figure 8, top). This occurs because there is a tendency in auditory streaming experiments (as reported in vision too, e.g., Mamassian and Goutcher, 2005) for the first phase to be much longer than subsequent phases (Denham et al., 2013); the short latency peak in the distribution corresponds to the initial responses (typically ABA-, A-, or B---), with the later peak corresponding to the first reports of percepts in subsequent phases.

The empirical cumulative distributions of latencies, which show on the y axis the proportion of latencies less than any given latency (in seconds) on the x axis (Figure 8, bottom), demonstrate the strong tendency for the *integrated* ABA- pattern to be reported with a far shorter latency than the other patterns, followed by similar latency distributions for the B---, A-, and -BA- patterns, and finally, much later the AB-- and A--- patterns. For example, by reading off the intercepts of each cumulative plot with the dashed red line we can see that 80% of first reports of ABA- occur within 5.5 s, of B--- within 120 s, -BA 204 s, A- 222 s, AB—308 s, and A--- 440 s. This may explain why the other patterns have not been reported in past experiments, as most of them have used sequences of short duration (typically <20 s). Wilcoxon rank sum test comparing cumulative distributions shows ABA- to be significantly different from all other patterns, *p* < 0.0001, B---, A-, and -BA- to be significantly different from ABA-, A---, and AB--, *p* < 0.0001, but not from each other (B---/-BA- *p* = 0.11, A-/-BA- *p* = 0.44, except B---/A- *p* = 0.013), and A--- and AB—to be significantly different from all other patterns, *p* < 0.0001, but not from each other, *p* = 0.098.)

#### Consistency of individual perceptual switching behavior

**Figure 9:**
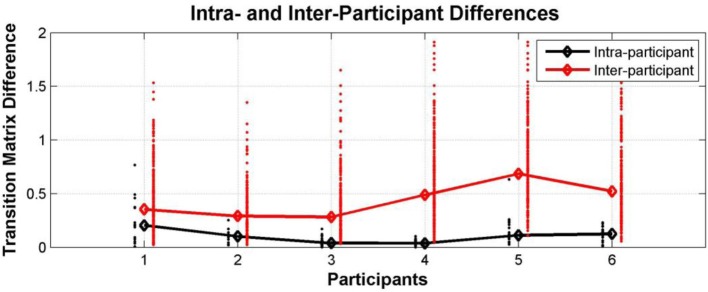
**Individual differences within Experiment 1**. Comparison between intra- and inter-individual perceptual switching behavior, characterized by participant transition matrices extracted for each participant for each test session, pooling data from all conditions; differences between transition matrices from the same participant at different sessions (black dots; median—black diamonds, line), differences between the transition matrices of each participant and those of all other participants (red dots; median—red diamonds, line).

The transition matrices constructed for each participant for each test session by pooling the data for all conditions were used to examine the consistency of individual behavior. Figure 9 below shows a comparison between intra- and inter-individual differences in terms of explicit difference measures. This demonstrates that individual participant behavior tends to be idiosyncratic; i.e., the switching behavior of an individual is more similar to their own behavior in a different test session than it is to the perceptual switching behavior of other participants (Wilcoxon rank sum test: participant 1 *p* = 0.0002, participants 2–6 *p* < 0.0001). It should be noted that by comparing the transition matrices of individual participants we go beyond just comparing switching rates; in addition to switching rates, the transition matrices capture the likelihood of reporting and switching between different perceptual patterns.

### Interim discussion

When listening to tone sequences of the form ABA-, participants report patterns other than the traditional integrated (ABA-) and segregated (A- or B---) ones if they are given the possibility to do so. Although the experiment is rather complex and participants require a number of training sessions before they are reliably able to perform the task, perceptual reports in the verification sections appended to the end of the stimuli provide confidence that participants were engaged in the task and accurately reported the patterns they perceived. Individual behavior was also consistent. We found that the perceptual switching behavior of each participant was very similar across sessions; median differences between an individual participant's transition matrices were small. In comparison, the differences between the transition matrices of different participants tended to be much larger; median differences were more than twice as large (except for participant 1, 1.8 times larger). This stable idiosyncratic behavior is interesting in its own right, as well as providing further confidence that participants were engaged in the task and were reliably reporting their perceptual experiences.

The choice of embedded patterns that we tested here was motivated in part by our modeling studies (Mill et al., 2013). The Chains model, which was used to successfully simulate the dynamics of the discovery and perceptual switching between the integrated and segregated organizations, can in principle also discover the embedded AB--, -BA-, and A--- patterns. Here we tested the perception of all repeating embedded patterns up to length 4. It is possible that listeners can spontaneously perceive even longer patterns if the sequence allows it, but we have not explored that possibility yet. Because we considered that participants may find the distinction too difficult to perceive, we did not ask participants to distinguish between the A--- and --A- patterns (i.e., complementary to -BA- and AB--, respectively), although the Chains model would predict that they are both perceived. The other limitation of the experimental design we used here is that we did not actually try to establish which perceptual organizations participants experienced; all we asked them to report was which pattern they perceived as dominant. On the basis of our theoretical proposals (Winkler et al., 2012) and the Chains model (Mill et al., 2013) we may infer the perceptual organizations, but these predictions remain to be properly tested in future experiments.

The distribution of the patterns other than *integration* and *segregation* (i.e., AB--, BA--, A---) in relation to the stimulus parameters, and the latency with which they are discovered, provide support for our hypothesis that the *both* response found in previous experiments (Bendixen et al., 2010; Denham et al., 2013) can be explained by the perception of embedded patterns not previously considered, rather than rapid switching between *integration* and *segregation*. The results are not directly comparable, as the participants and range of stimulus parameters tested were somewhat different. However, qualitatively the relationship between stimulus parameters and the probability of reporting *both* seems to be well explained by a combination of the AB--, -BA-, and A--- patterns. *Both* is most commonly reported for small frequency differences and fast presentation rates, and is less common when the stimulus parameters strongly promote *segregation* or *integration* (Denham et al., 2013). Furthermore, *both* was hardly ever reported as the first percept (Denham et al., 2013). Here the median latency for reporting the AB--, -BA-, and A--- patterns tended to be rather long, and in all conditions in this experiment the median latency for at least one of the set {ABA-, A-, B---} was always less than the minimum median latency for the set {AB--, -BA-, A---}. These factors lead us to believe that the *both* response in previous experiments corresponds to the dominant perception of one of the following patterns AB--, -BA-, or A---.

It could be argued that participants only perceived all the patterns reported here because they were trained to do so, and that without this training they would not have heard patterns such as AB--, -BA-, or A---. There are a number of factors that argue against this objection. Firstly, in a pilot experiment (reported in Denham et al., 2013), listeners were asked to verbally report all repeating patterns that they experienced during a 4-min long ABA- sequence. Most of the patterns trained in the current experiment were described spontaneously by listeners in this pilot study. Secondly, in our previous experiments *both* was reported without extensive pattern-specific prior training. Thirdly, the proportion and latency characteristics of *both* responses correspond well to those of the grouped {AB--, -BA-, A---} responses. On this basis, we argue here that *both* corresponds to one or other of the patterns AB--, -BA-, or A---. Finally, participants were instructed to adopt a neutral listening approach and not attempt to hear one or other pattern. Therefore, we would argue that the influence of training on the reports of these other patterns was largely limited to facilitating their categorization and reporting, rather than increasing their incidence.

Experiment 1 raised two important questions that we investigated in two follow-up experiments. Firstly, we decided to probe the stability of individual consistency in perceptual switching behavior and individual differences between participants over a longer time scale. If these are truly individual differences (i.e., differences of a physical nature), we would expect to find that the perceptual switching behavior of participants is similar even when tested in sessions separated by rather long periods of time, and that the individual differences found here also remain reliably detectable. Secondly, we were surprised by the relative prominence of the B--- pattern relative to the A- pattern. If anything the Chains model would predict that the A- pattern should be more prominent as it occurs more often (in Chains terms, makes more successful predictions per unit time) than the B--- one does. We hypothesized that the higher frequency of the B tones relative to the A tones in all conditions may have made the B tones perceptually more salient, and that this was the cause of the higher prominence of the B--- pattern. For these reasons we conducted two recall experiments (Experiments 2 and 3) approximately 1 year after Experiment 1.

## Experiment 2

### Introduction

Individuals are known to differ markedly in their perceptual behavior in visual multistability experiments; e.g., individual differences in perceptual switching rate in binocular rivalry have been known for many years (Aafjes et al., 1966). More recently, genetic markers (Miller et al., 2010) and differences in brain structure (e.g., Kanai et al., 2010; Genc et al., 2011), have been associated with differences in typical individual switching rates. Biases toward different perceptual decisions have also been reported and shown to relate, in the case of bistable motion, to differences in inter-hemispheric connectivity (Kanai and Rees, 2011). However, although there are some pointers toward such differences in audition (e.g., see Kondo and Kashino, 2009; Kashino and Kondo, 2012), a systematic investigation of the genetic or physiological basis for differences in auditory perceptual multistability has not yet been attempted. In Experiment 2, we sought to investigate whether the individual differences found in Experiment 1 were stable over a prolonged period. We hypothesized that if, as in vision, auditory individual perceptual differences are a result of stable physiological or even genetic differences, then we should find that the individual differences in perceptual switching behavior reported in Experiment 1 would be detectable 1 year later.

### Methods

#### Participants

Five (mean age 23.2 years, range 20–26 years; all right-handed; 3 male, 2 female) of the original six participants took part in Experiment 2, which was conducted over four sessions approximately 1 year after Experiment 1.

All equipment and procedures were as described for Experiment 1.

#### Training procedure

Participants attended one training session, which was similar in form to the training sessions for Experiment 1.

#### Testing procedure

Participants attended three test sessions over a duration of 2 weeks with at least 2 days between consecutive sessions during which the five 10-min experimental blocks were presented. All instructions and procedures were as for Experiment 1.

#### Analysis

To compare the effects of experiment (i.e., comparing Experiment 1 with the recall Experiment 2) we used a Two-Way repeated-measures ANOVA with two factors, experiment comparison (2 levels: same vs. other) × participant comparison (2 levels: self vs. other). For each of the 4 (2 × 2) comparisons we calculated the mean of all possible KL distances between the corresponding transition matrices. The ANOVA was computed on the resulting 20 values, i.e., 5 participants × 2 experiment levels × 2 participant levels.

### Results

From the entire data set for Experiment 2 the removal of phases where no button was pressed or duration was less than 300 ms resulted in the removal of 5.1% of the total data.

**Figure 10:**
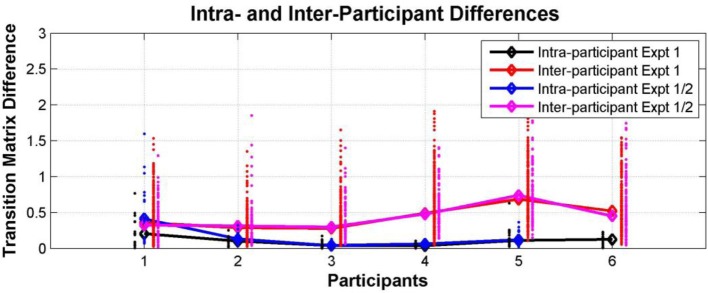
**Individual differences within and between Experiments 1 and 2**. Comparison between intra- and inter-individual perceptual switching behavior, characterized by participant transition matrices extracted for each participant for each test session, pooling data from all conditions. Differences between transition matrices from the same participant in different sessions in Experiment 1 (black dots; median—black diamonds, line), differences between transition matrices of each participant in Experiment 1 vs. sessions from the same participant in Experiment 2 (blue dots; median—blue diamonds, line), differences between the transition matrices of each participant and those of all other participants in Experiment 1 (red dots; median—red diamonds, line), and differences between the transition matrices of each participant from Experiment 1 and those of all other participants in Experiment 2 (magenta dots; median—magenta diamonds, line).

Participant transition matrices were constructed for each participant for each test session. Figure 10 compares intra- and inter-individual differences in perceptual switching behavior in Experiments 1 and 2. The ANOVA analysis showed that the effect of participant (self vs. other) was highly significant [*F*_(1, 16)_ = 20.49, *p* = 0.0003], while the effect of experiment (1 vs. 2) was not significant [*F*_(1, 16)_ = 0.38, *p* = 0.55], and there was no interaction between these factors [*F*_(1, 16)_ = 0.06, *p* = 0.81].

### Interim discussion

Individual differences in perceptual switching behavior remained consistent and detectable a year after the first experiment. All of the participants behaved in a way that was more similar to their behavior at all other sessions, including those separated by a year from each other, than to any of the other participants. This internal consistency over such a long period suggests that switching patterns in the current multistable auditory paradigm reflect some stable perceptual or higher-level traits, possibly stemming from physiological and maybe even genetic differences between listeners.

## Experiment 3

### Introduction

In Experiment 3 we investigated whether the frequency relation of the two tones (A lower than B, or *vice versa*) could influence the extent to which the A- and B--- patterns were reported as the foreground pattern by listeners. In Experiment 1 the frequency of the A tones was 400 Hz, and that of the B tones 476, 599, or 1008 Hz. Equal loudness curves of normal listeners (e.g., see Moore, 2003), suggest that the B tones would be perceptually louder than the A tones, and thus more salient. Therefore, in this experiment we decided to test whether switching the frequencies of the A and B tones would result in a greater tendency for listeners to report the A- than the B--- pattern.

### Methods

#### Testing

The same five participants as in Experiment 2 took part in three “reverse frequency” test sessions in Experiment 3. Stimuli were arranged in five conditions, with frequency difference (Δ*f*) and stimulus onset asynchrony (SOA) as follows: (1) Δ*f* = 3 st, SOA = 100 ms; (2) Δ*f* = 16 st, SOA = 100 ms; (3) Δ*f* = 7 st, SOA = 150 ms; (4) Δ*f* = 3 st, SOA = 200 ms; (5) Δ*f* = 16 st, SOA = 200 ms. The “A” and “B” tones were delivered with a common duration of 75 ms (including 10 ms rise and fall times). The frequency of the “B” tones was 400 Hz, and the frequency of the “A” tones was *n* semitones higher, depending on condition. Participants were presented with all five conditions in a randomized order at each test session.

#### Analysis

To analyse the effect of changing the stimulus parameters (exchanging the A and B tone frequencies) a Two-Way repeated-measures ANOVA with factors experiment (1/3) and condition (1–5) was conducted. The dependent measure was the difference between the proportion of B--- and A- reported by each participant for each condition in Experiments 1 and 3. *Post-hoc* Wilcoxon rank sum tests were used to compare the effect of experiment in each condition separately. To analyse whether intra-individual similarities and inter-individual differences were preserved in Experiment 3, we performed the same Wilcoxon rank sum tests used for Experiment 1 to compare the distributions of intra- vs. inter-individual differences for each participant separately.

### Results

From the entire data set for Experiment 3 the removal of phases where no button was pressed or duration was less than 300 ms resulted in the removal of 5.3% of the total data.

**Figure 11:**
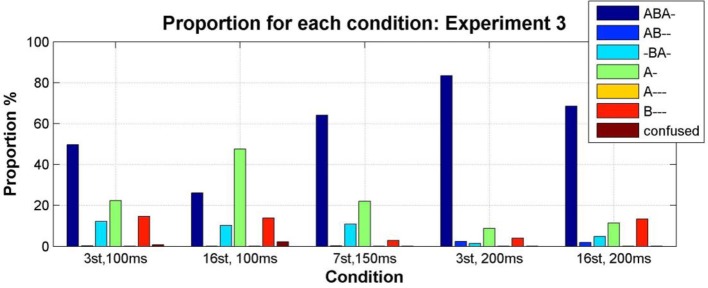
**Proportion of the total stimulus duration during which each pattern was perceived (color coded as indicated by the accompanying legend) for each condition, calculated from the condition transition matrices constructed by pooling the data from all participants for each condition (Bõhm et al., 2013)**.

Condition transition matrices were constructed as described for Experiment 1, and used to plot the mean proportion of each pattern as a function of condition. As predicted, exchanging the frequencies of the A and B tones resulted in the A- pattern becoming more prominent than the B--- pattern; see Figure 11 in comparison with Figure 5 (a repeated-measures Two-Way ANOVA with factors experiment (1/3) and condition (1–5) showed a significant effect of experiment on the difference between the proportion of B--- and A-, *F*_(4, 40)_ = 16.75, *p* = 0.0002. The interaction between experiment and condition was also significant, *F*_(4, 40)_ = 3.85, *p* = 0.0097. This was caused by the significant effect of experiment in condition 2 (Wilcoxon rank sum test *p* = 0.03), and no significant effect of experiment in other conditions (Wilcoxon rank sum test *p* > 0.15 for conditions 1, 3, 4, and 5).

**Figure 12:**
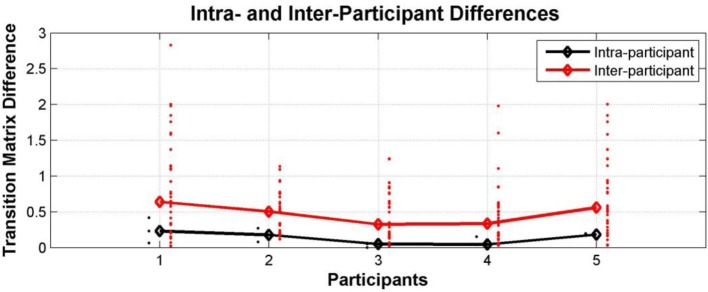
**Comparison between intra- and inter-individual perceptual switching behavior in Experiment 3; differences between transition matrices from the same participant at different sessions (black dots; median—black diamonds, line), and differences between the transition matrices of each participant and those of all other participants (red dots; median—red diamonds, line)**.

We also examined intra- and inter- individual differences in Experiment 3 by constructing participant transition matrices as described in Experiment 1. As shown in Figure 12, these characteristics are still present when the frequencies are reversed; i.e., once more we find for all participants that, the switching behavior of each individual is more similar to their own behavior in a different test session than it is to the perceptual switching behavior of other participants (Wilcoxon rank sum test: *p* = 0.02, 0.008, 0.0003, 0.001, 0.007 for participants 1–5, respectively).

### Interim discussion

Exchanging the A and B tone frequencies led to increased prominence of the A- pattern and decreased prominence of the B--- pattern. This supports the idea that perceptual saliency of the A and B tones (determined, amongst other factors, by their relative perceived loudness) can cause one or other of these patterns to become more dominant, i.e., occupy the perceptual foreground. This provides further support for a competition account of auditory streaming (Winkler et al., 2012), and suggests that models of auditory scene analysis should also take account of the influence of event saliency on perceptual organization.

## General discussion

The experiments presented here provide support for the hypothesis that auditory perception involves the extraction of patterns (regularities) from incoming sequences of sound events and that multiple patterns can be detected and held in parallel. Even when listening to simple repeating ABA- sequences, participants reported up to six different foreground patterns. This suggests that given sufficient time, pattern discovery appears to be exhaustive, i.e., all possible patterns within a certain length are perceived. We did not attempt to investigate whether there is a limit on the length of the patterns that are discovered and used by the auditory system to parse the auditory scene; this remains to be explored, probably with more complex sequences. However, our inclusion of patterns up to length four is supported by related ERP studies (Boh et al., 2011).

Once patterns have been discovered they come and go from conscious perception for as long as the stimulus sequence continues. This is consistent with the proposal that the contents of perceptual awareness are the result of an on-going competitive process between the set of patterns that have been discovered (Winkler et al., 2012). The distribution of these patterns in relation to the stimulus parameters, and the latency with which they were reported in Experiment 1 leads us to conclude that the *both* reports in previous experiments are consistent with the perception of foreground patterns AB--, -BA-, or A---. Based on our modeling studies we infer that the background pattern perceived in each of these cases was --A-, A---, and -BA-, respectively, although we did not attempt to investigate the perception of background patterns here.

Although bi-/multi-stable perceptual switching is highly stochastic, the switching patterns of individuals could be distinguished from each other. This is the first time to our knowledge that intra-individual similarities and inter-individual differences have been documented for *patterns* of perceptual switching in auditory streaming. However, individual differences in the number of perceptual switches have been previously reported (Kondo et al., 2012), and in vision individual differences in binocular rivalry have been known for some time (Aafjes et al., 1966). The method we use to distinguish individuals is different from the switching rate measure that has previously been used. Here, we characterized the difference between two individuals in terms of a single distance measure between their transition matrices. However, much remains to be investigated regarding the details of these differences, which are likely to stem from some combination of switching rate, perceptual biases in the proportion of the various patterns perceived, and perhaps even higher order relationships such as idiosyncratic perceptual transitions.

The finding that individuals behave in a measurably consistent manner even between sessions separated by a year leads us to suggest that relatively stable bases such as anatomical or genetic differences, similar to those found for other multi-stable phenomena (Kanai et al., 2010; Genc et al., 2011), may be responsible. However, the neural correlates of perceptual dominance and perceptual switching in auditory perception are not yet well understood, although there is some reason to suppose that there may be some aspects that are shared with vision (e.g., Cusack, 2005). We suggest that the paradigm and analysis methods we present here may prove useful in the future for investigations of the neural basis for auditory perceptual organization.

Another question of interest for future investigations is whether, and if so what, other individual perceptual or cognitive characteristics are related to these individual patterns of switching behavior. If our assumption regarding the mechanisms underlying auditory perception are correct (i.e., discovery and on-going competition between alternative interpretations of the input), then these individual differences may underlie variation in other characteristics, such as perceptual abilities, cognitive style, personality or creativity.

The results of these experiments have implications for models of auditory scene analysis; in particular, any comprehensive model should account for the multitude of patterns that participants report, the parameter dependence of the pattern distributions, and the latencies with which they are discovered. No model has been developed yet which can account for all of these aspects. The popular temporal coherence model in its current formulation makes a fixed perceptual decision (e.g., Shamma et al., 2011). While this could undoubtedly be modulated by the introduction of noise and adaptation, it is more difficult to see how the other embedded patterns reported here, e.g., -BA- could be discovered, as the temporal coherence measure would either group or not group A's with B's. The Chains model (Mill et al., 2013) in its current formulation cannot discover the AB--, -AB-, or A--- patterns reported here either, but this is easily fixed with a simple modification to the pattern discovery function. However, there is a more fundamental problem; since the links in Chains form probabilistically between events, patterns involving two events, e.g., AB--, will be easier to discover than patterns involving three events, ABA-. Therefore, Chains would predict that AB-- is reported with a shorter latency than ABA-, which is not the case.

## Conclusion

Although on the face of it the results we present here appear to challenge our everyday experience of perception as stable and veridical, we suggest that it is precisely by having the ability to construct multiple interpretations of a scene that perception is able to achieve robust performance. The first reported pattern corresponds to the most likely interpretation; here, typically that there is one object in the world producing sounds of different pitch but similar timbre, although if the frequency difference becomes too great then the possibility of two sound sources becomes more likely. It is important to note that this initial perceptual decision is not simply a function of the physical stimulus; relevant contextual information (Snyder et al., 2008, 2009b) and prior learning (van Zuijen et al., 2005; Snyder et al., 2009a) also exert an influence. It also makes sense that perception never fixes on a single solution. If either of these were the case then our perceptions would be entirely determined by external factors, leaving no room for autonomous behavior. Since perception is essentially about trying to extract information from the world around us, a better strategy than simply choosing the “best” interpretation is to explore other interpretations if time allows in case they offer insights not available in the most likely scenario. By setting up a competition between alternatives and ensuring that no solution can dominate forever, the perceptual system essentially performs a probabilistic likelihood sampling of the perceptual space (Moreno-Bote et al., 2011). This view of perception resonates with a number of earlier perceptual theories, including Helmholtz's view of the role of inferential processes in perception (Helmholtz, 1885), Gregory's notion of perception as hypotheses (Gregory, 1980), and more recent instantiations in work on predictive coding theory by Friston and colleagues amongst others (e.g., Friston and Kiebel, 2009). Competition between patterns, rather than individual elements, also fits well with Gestalt ideas of perception that emphasize the importance of the whole (i.e., patterns) relative to the parts (i.e., individual tones) (Köhler, 1947; Wagemans et al., 2012). In conclusion, studies of perceptual multistability can provide new and useful insights into general mechanisms as well as individual differences in human perception.

### Conflict of interest statement

The authors declare that the research was conducted in the absence of any commercial or financial relationships that could be construed as a potential conflict of interest.
