# Supplementary material for: Stable individual characteristics in the perception of multiple embedded patterns in multistable auditory stimuli
Source: Front Neurosci. 2014 Feb 28;8:25. doi: 10.3389/fnins.2014.00025 (PMC3937586; doi:10.3389/fnins.2014.00025)
Supplement: Supplementary file 1 [file DataSheet1.PDF]

Supplementary Analysis: Stable individual characteristics in the perception of multiple embedded patterns in multistable auditory stimuli

---

Individual differences: Experiment 1

The following analysis explores to what extent the following factors can characterise individual differences in multistable perception: latency to first report, proportion of all patterns, proportion ABA-, proportion AB--, proportion –BA-, proportion A-, proportion B---, number of switches. For comparison we include the KL differences reported in the paper. For ease of comparison non-significant p values, i.e. indicating that the individual is not distinguishable from the group, are highlighted in red.

1. **KL differences**

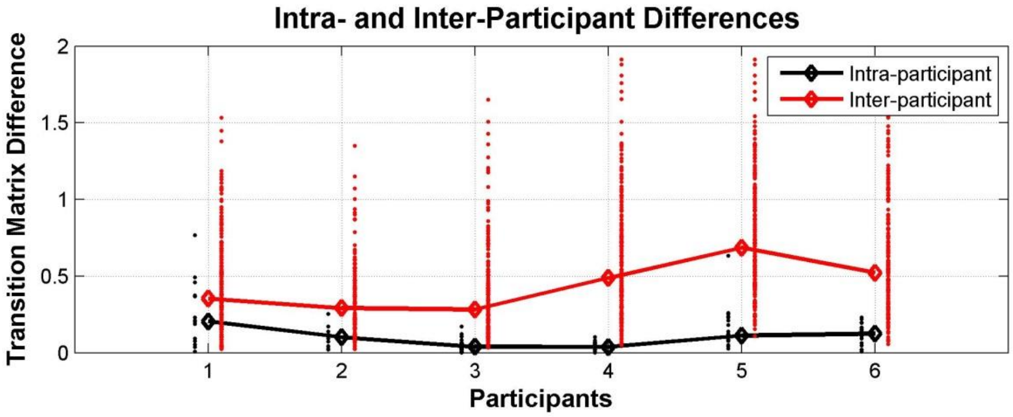

p=0.000211, 0.000000, 0.000000, 0.000000, 0.000000, 0.000000 for participants 1-6, respectively.

2. **Latency to first report**

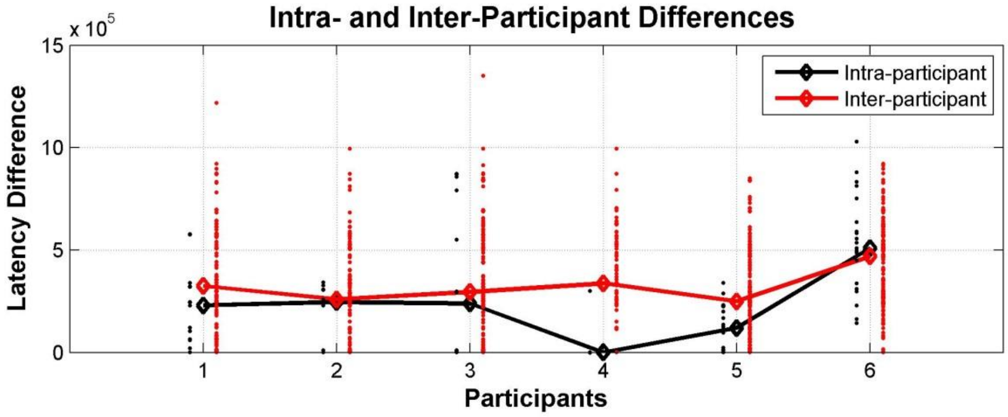

p=0.000884, 0.005523, 0.002922, 0.000000, 0.000268, 0.913844

Calculated as sum-squared difference between latency vectors

3. **Proportions of patterns (pattern over all)**

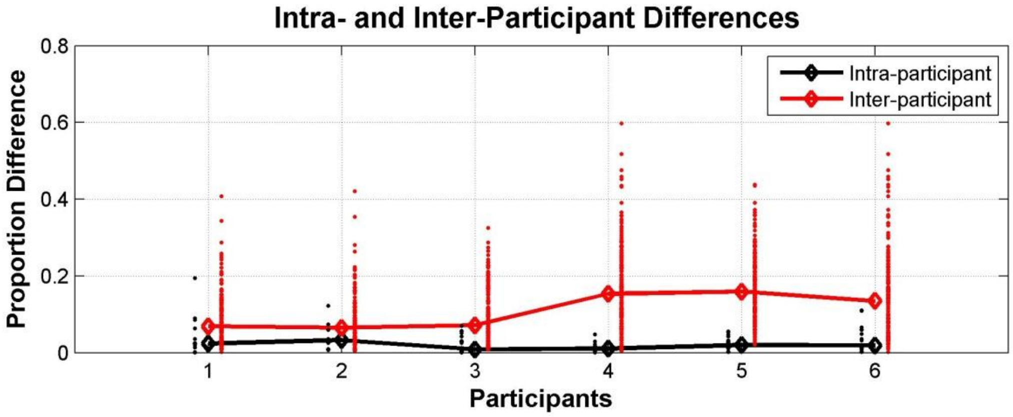

p=0.006428, 0.275836, 0.054566, 0.000000, 0.000011, 0.030561

Calculated as sum-squared difference between proportion vectors

4. **Proportion ABA-**

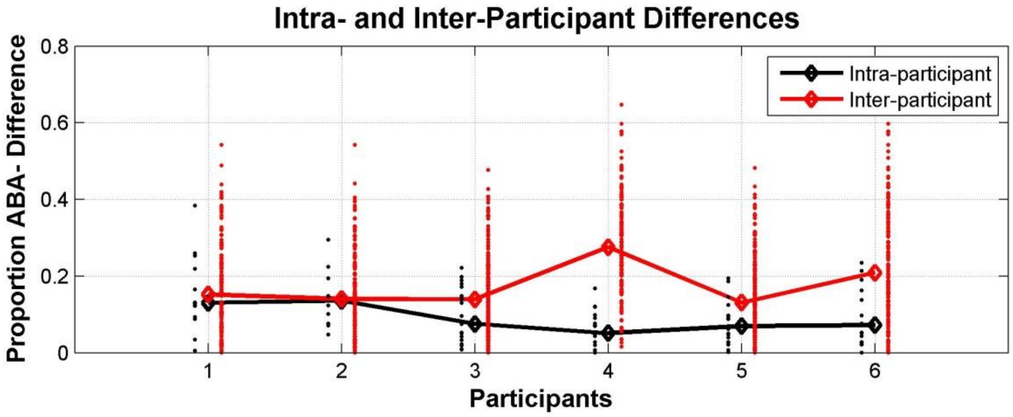

p=0.873744, 0.739022, 0.001185, p=0.000000, p=0.000013, p=0.000000

Calculated as absolute difference between proportions.

5. **Proportion AB--**

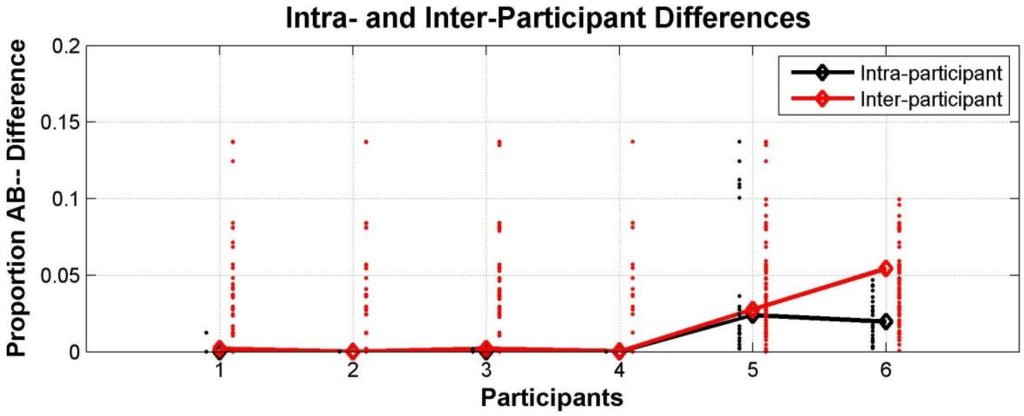

p=0.002714, 0.009964, 0.000207, 0.000000, 0.437152, 0.000000

Calculated as absolute difference between proportions.

6. **Proportion -BA-**

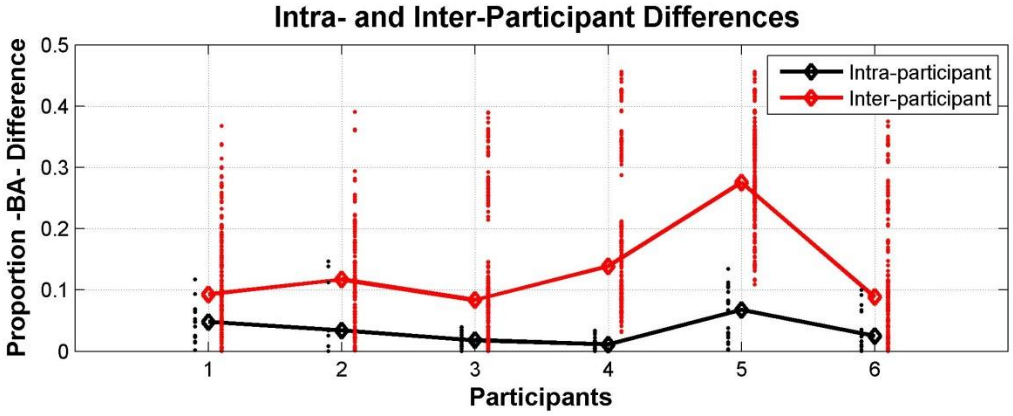

p=0.000001, 0.003037, 0.000000, 0.000000, 0.000000, 0.000000

Calculated as absolute difference between proportions.

7. **Proportion A-**

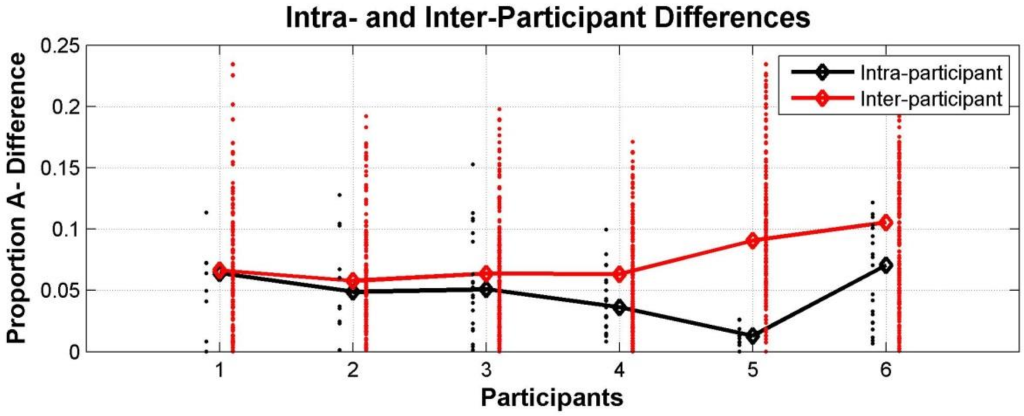

p=0.122830, 0.613491, 0.188614, 0.000504, 0.000000, 0.000014

Calculated as absolute difference between proportions.

8. **Proportion B---**

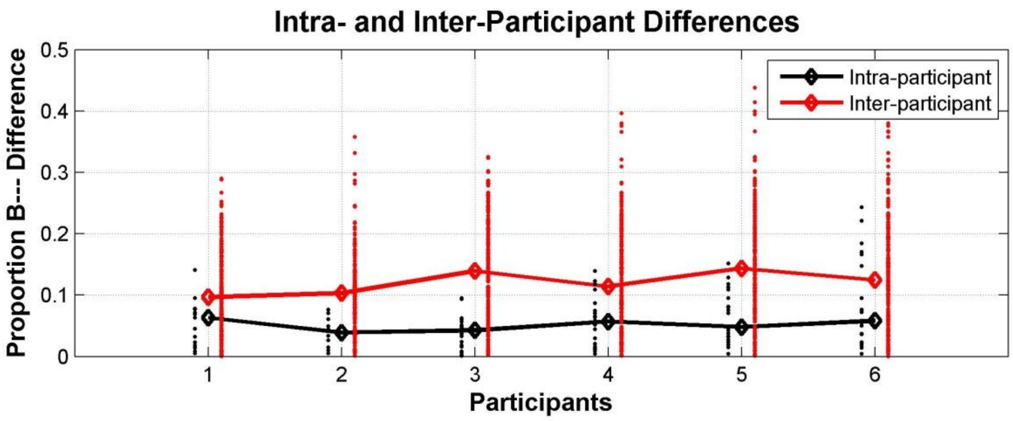

p=0.000075, 0.000002, 0.000000, 0.000000, 0.000000, 0.001629

Calculated as absolute difference between proportions.

9. **Number of switches**

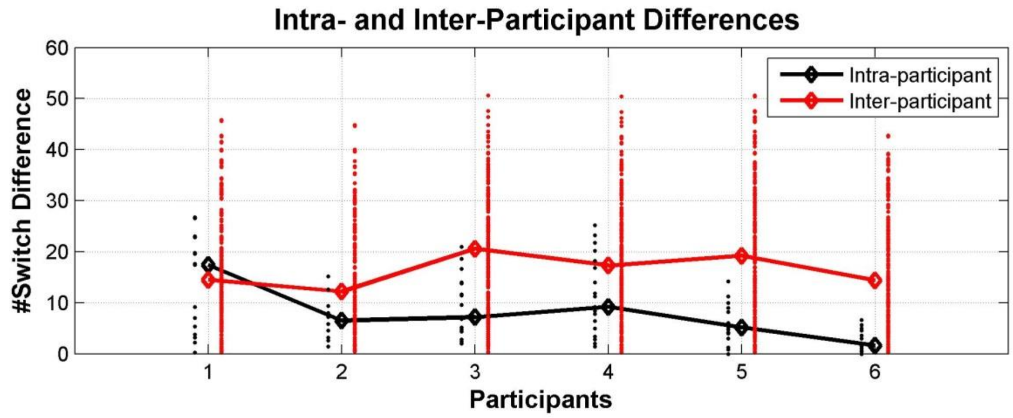

p=0.237735, 0.001273, 0.000000, 0.000123, 0.000000, 0.000000

Calculated as absolute difference between number of switches.

Conclusion

As suggested by reviewer 2, there are specific characteristics of perceptual switching which can be used to characterise individual differences; all of which are subsumed within the transition matrix representation.

Stability of individual differences: Experiment 1 + 2

The following analysis explores to what extent the factors analysed above are stable over the period of a year separating experiments 1 and 2. For comparison we include the KL differences reported in the paper. For ease of comparison non-significant p values, i.e. indicating that the individual is not distinguishable from the group, are highlighted in red.

1. **KL differences**

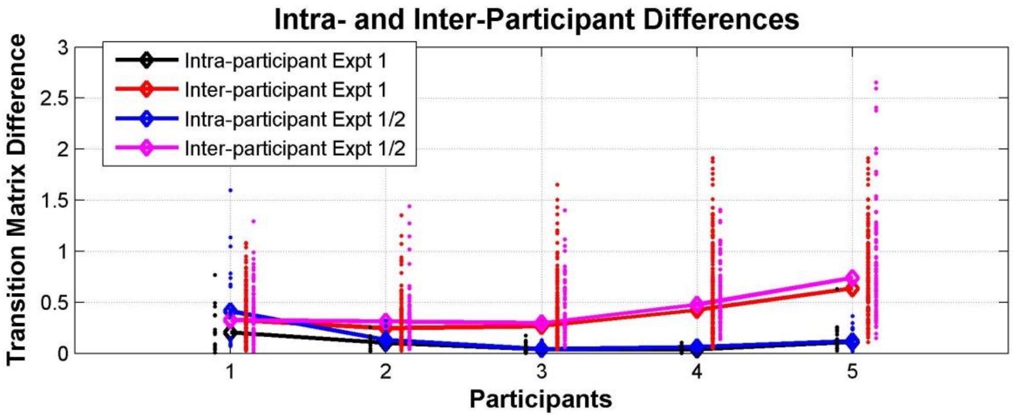

ANOVA: Self vs. other is highly significant (F(1,16)=20.49, p=0.0003); effect of experiment (1 vs. 2) is not significant (F(1,16)=0.38, p=0.55), and there is no interaction between these factors (F(1,16)=0.06, p=0.81).

2. **Latency to first report**

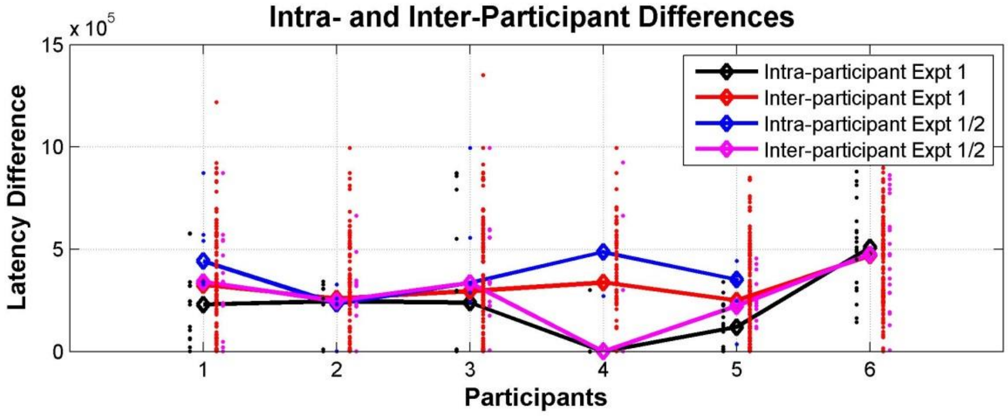

ANOVA: Self vs. other is not significant (F(1,16)=3.01, p=0.10); effect of experiment (1 vs. 2) is not significant (F(1,16)=0.53, p=0.48), and there is no interaction between these factors (F(1,16)=0.87, p=0.37).

3. **Proportions of patterns (pattern over all)**

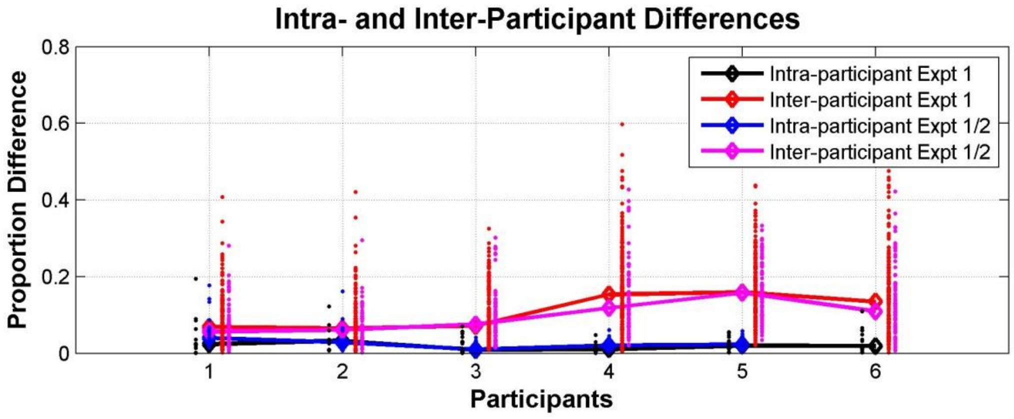

ANOVA: Self vs. other is highly significant (F(1,16)=31.53, p<0.0001); effect of experiment (1 vs. 2) is not significant (F(1,16)=0.07, p=0.8), and there is no interaction between these factors (F(1,16)=0.26, p=0.62).

4. **Proportion ABA-**

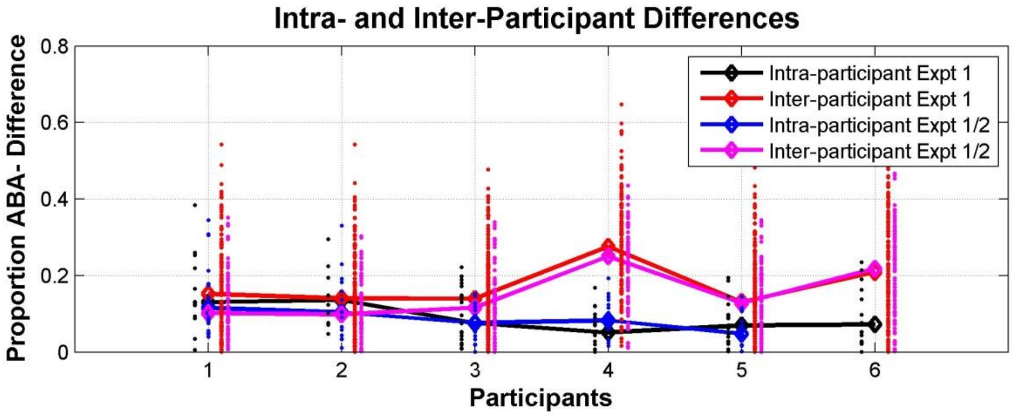

ANOVA: Self vs. other is significant (F(1,16)=9.79, p=0.0065); effect of experiment (1 vs. 2) is not significant (F(1,16)=1.15, p=0.3), and there is no interaction between these factors (F(1,16)=0.28, p=0.6).

5. **Proportion AB--**

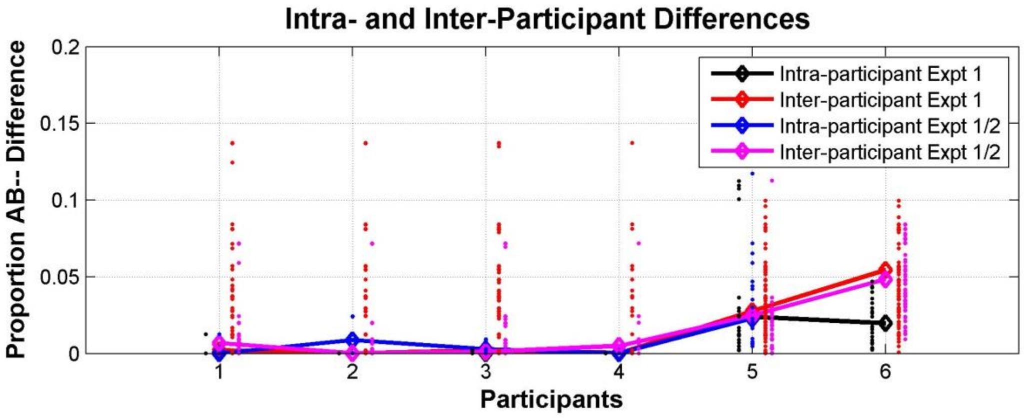

ANOVA: Self vs. other is not significant (F(1,16)=2.5, p=0.13); effect of experiment (1 vs. 2) is not significant (F(1,16)=0.35, p=0.56), and there is no interaction between these factors (F(1,16)=0.58, p=0.46).

6. **Proportion -BA-**

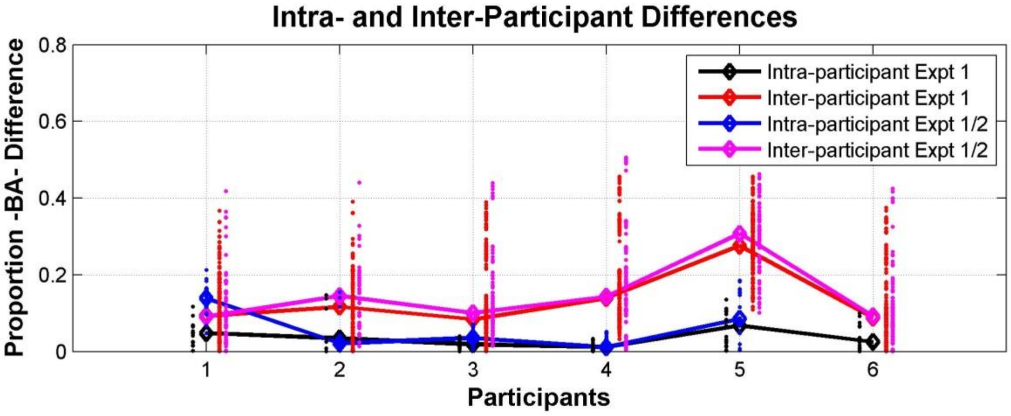

ANOVA: Self vs. other is significant (F(1,16)=19.77, p=0.004); effect of experiment (1 vs. 2) is not significant (F(1,16)=0.43, p=0.52), and there is no interaction between these factors (F(1,16)=0.08, p=0.79).

7. **Proportion A-**

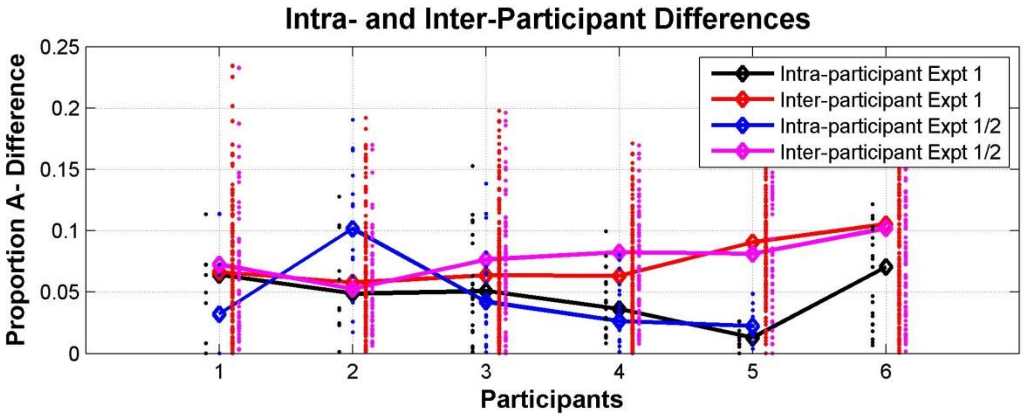

ANOVA: Self vs. other is significant (F(1,16)=10.63, p=0.0049); effect of experiment (1 vs. 2) is not significant (F(1,16)=0.17, p=0.68), and there is no interaction between these factors (F(1,16)=0, p=0.95).

8. **Proportion B---**

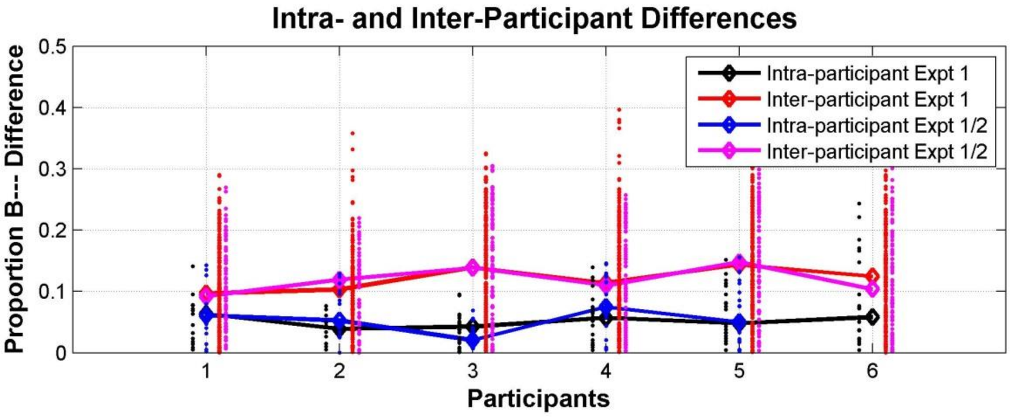

ANOVA: Self vs. other is significant (F(1,16)=76.65, p<0.0001); effect of experiment (1 vs. 2) is not significant (F(1,16)=0.22, p=0.65), and there is no interaction between these factors (F(1,16)=.45, p=0.51).

9. Number of switches

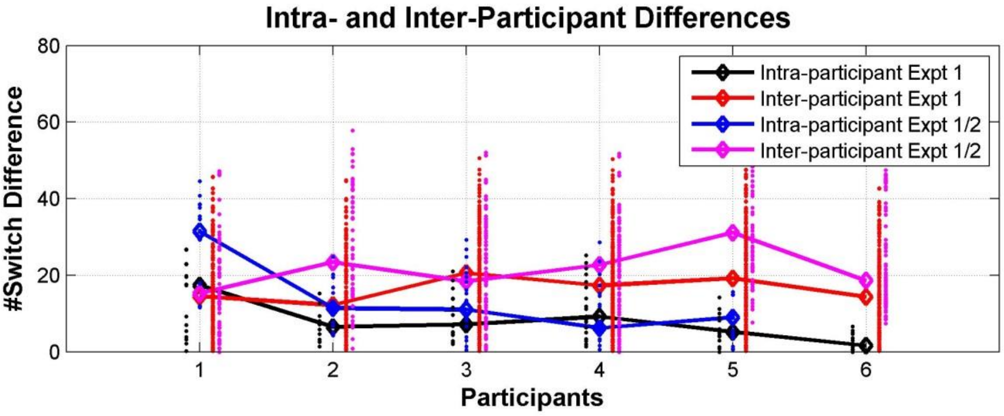

ANOVA: Self vs. other is significant (F(1,16)=14.06, p=0.0017); effect of experiment (1 vs. 2) is significant (F(1,16)=4.69, p=0.0457), and there is no interaction between these factors (F(1,16)=.04, p=0.84).

Conclusion

The transition matrix KL difference and the pattern of proportions (and in this case specifically the proportion of –BA- and B---) are most stable over time, while the latency for each pattern and the total number of switches are not.

Individual differences: Experiment 3

The following analysis explores to what extent the factors analysed above are affected by a change in stimulus parameters. For comparison we include the KL differences reported in the paper. For ease of comparison non-significant p values, i.e. indicating that the individual is not distinguishable from the group, are highlighted in red.

1. **KL differences**

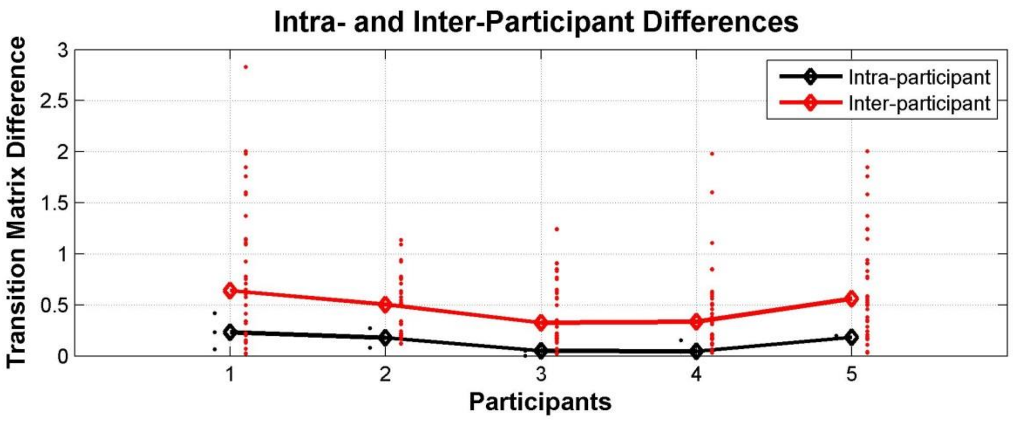

p=0.022446, p=0.008236, 0.000348, 0.001294, 0.006645

2. **Latency to first report**

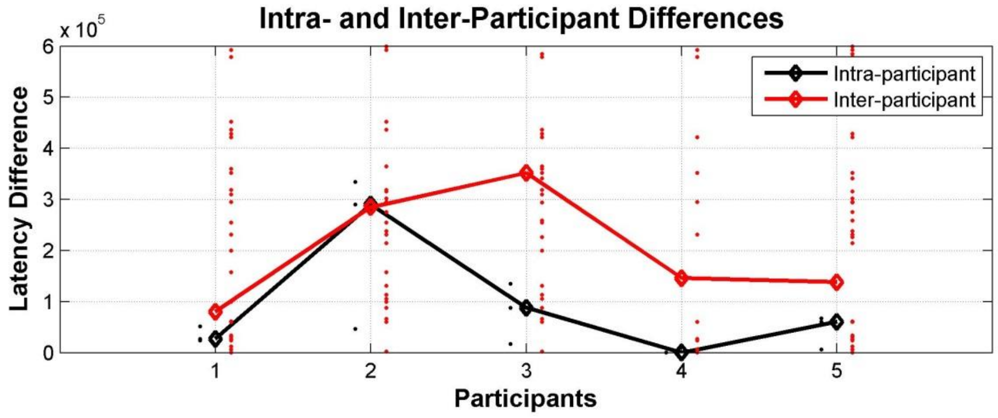

p=0.135734, 0.577388, 0.002114, 0.000111, 0.288924

3. **Proportions of patterns (pattern over all)**

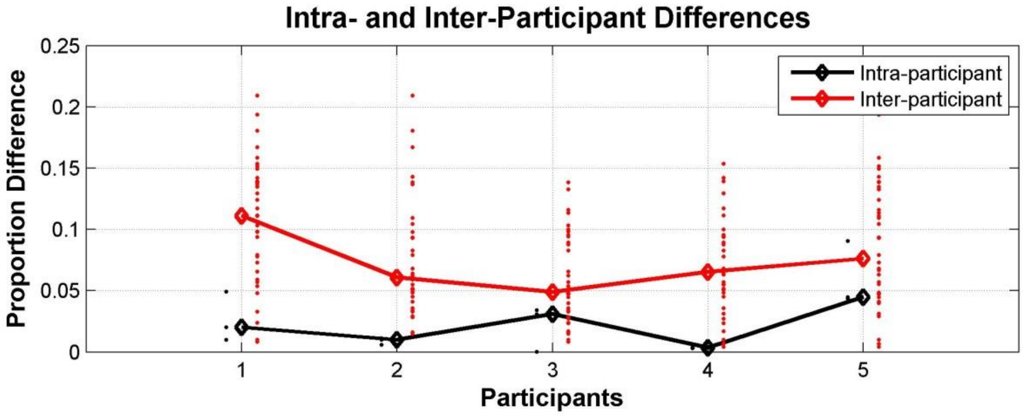

p=0.001294, 0.000111, 0.015244, 0.000149, 0.155616

4. **Proportion ABA-**

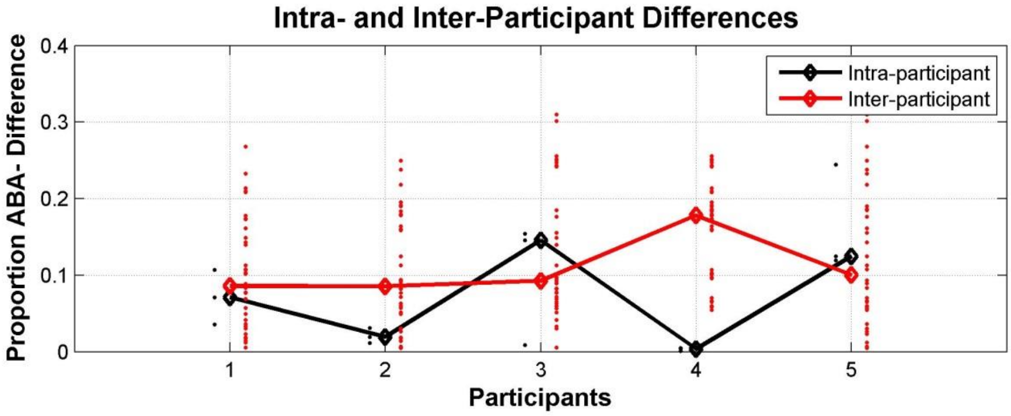

p=0.483304, 0.008236, 0.787457, 0.000111, 0.177635

5. **Proportion AB--**

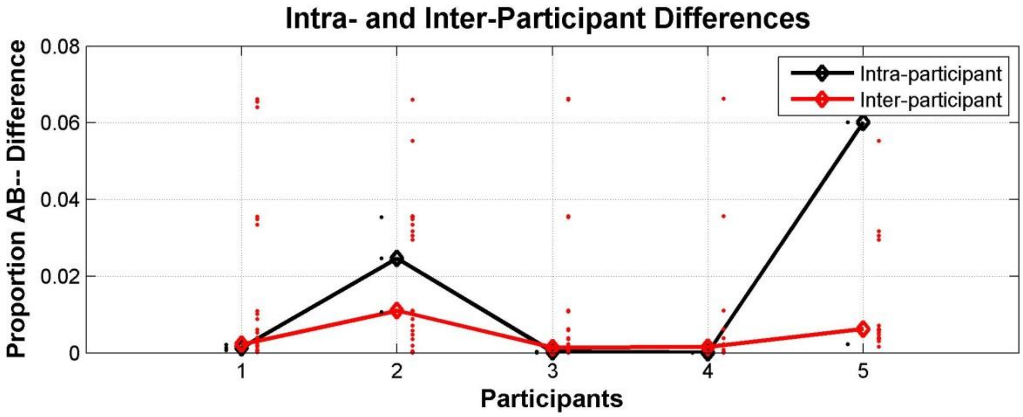

p=0.322865, 0.483304, 0.064119, 0.002685, 0.956997

6. **Proportion -BA-**

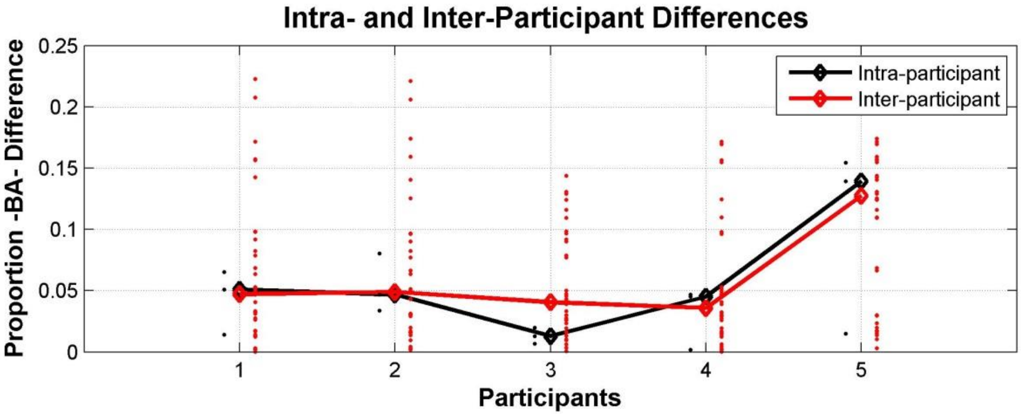

p=0.956997, 0.732718, 0.006645, 0.439584, 0.577388

7. **Proportion A-**

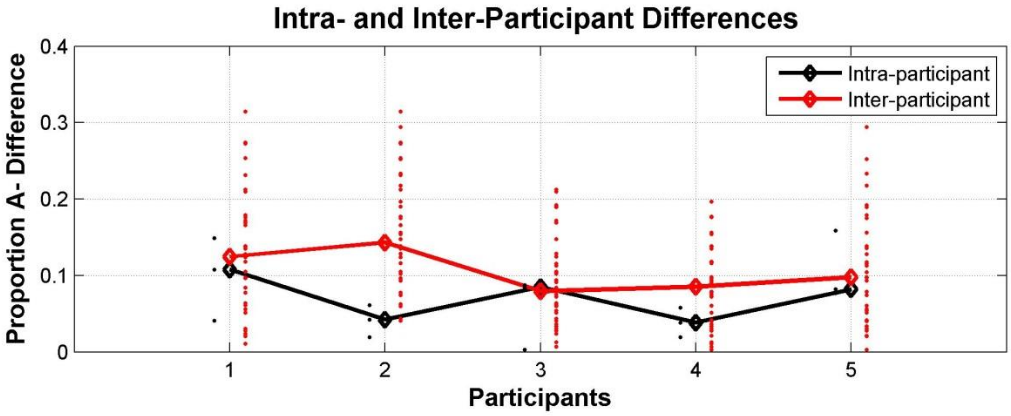

p=0.577388, 0.000348, 0.257474, 0.038730, 0.732718

8. **Proportion B---**

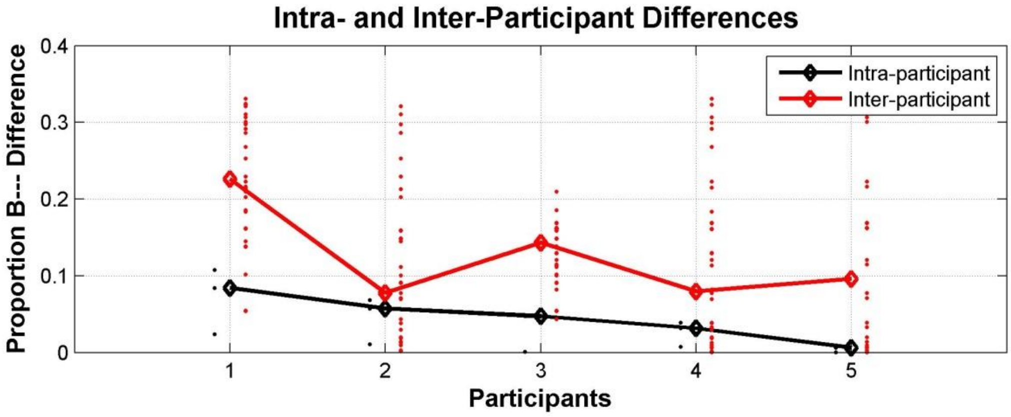

p=0.000457, 0.135734, 0.000199, 0.101910, 0.001658

9. Number of switches

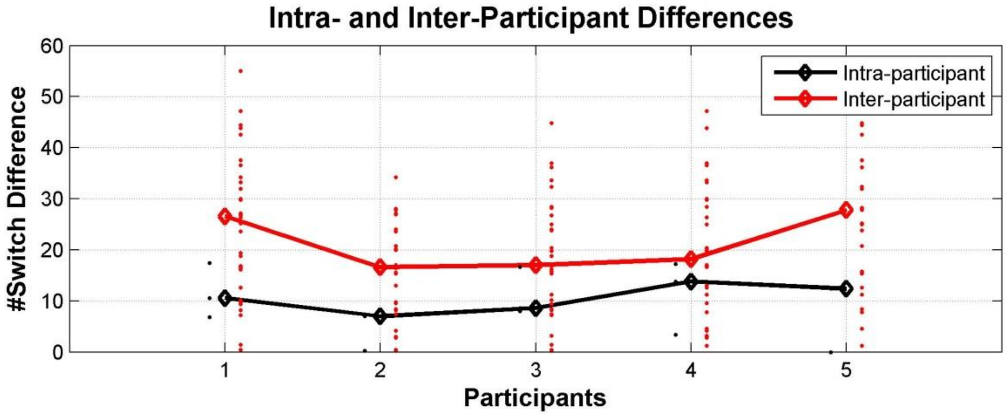

p=0.027028, 0.001654, 0.135639, 0.228425, 0.004233

Conclusion

Although there are many characteristics of perceptual switching which can be used in an ad hoc way to characterise individual differences, by considering experiment 3 here as well, it seems that the KL differences between transition matrices provides the most consistent way of doing so. This may be because the factors that distinguish people depend to some extent on the set of people and on the experimental conditions. Therefore, a method which integrates the individual factors into a unified measure provides the most reliable way for distinguishing between individuals.

Effect of emphasized pattern on next block

In experiment 1 there are 37 examples of non-integrated first phases; details below. For each the previous emphasized pattern was extracted using our training records.

Pattern numbering: 1. ABA-, 2. AB--, 3. –BA-, 4. A-, 5. A---, 6. B---, 7. confused

Participant	Condition	Session	Reported Pattern	Duration	Previous emphasized pattern
1.0000	2.0000	5.0000	6	94.7560	6
1.0000	2.0000	6.0000	4	110.8320	6
1.0000	5.0000	6.0000	4	7.4280	6
1.0000	1.0000	7.0000	3	21.2440	4
1.0000	2.0000	7.0000	4	18.6920	6
1.0000	2.0000	9.0000	6	50.7560	6
1.0000	2.0000	10.0000	6	13.0640	6
1.0000	2.0000	11.0000	6	10.7000	6
2.0000	2.0000	5.0000	6	62.7480	1
2.0000	3.0000	6.0000	2	10.9640	4
2.0000	2.0000	9.0000	6	32.5320	6
2.0000	2.0000	11.0000	6	22.9480	4
3.0000	2.0000	4.0000	6	30.6720	1
3.0000	2.0000	5.0000	6	19.1040	first block
3.0000	2.0000	6.0000	6	76.7200	6
3.0000	2.0000	7.0000	6	7.3720	2
3.0000	2.0000	8.0000	6	16.2200	2
3.0000	2.0000	10.0000	6	119.6320	2
4.0000	2.0000	5.0000	4	1.8520	4
4.0000	2.0000	8.0000	4	2.1000	4
5.0000	2.0000	4.0000	6	31.0920	2
5.0000	2.0000	5.0000	4	14.7760	2
5.0000	2.0000	6.0000	6	31.2200	4
5.0000	2.0000	8.0000	6	21.2440	2
5.0000	2.0000	9.0000	6	56.9480	3
5.0000	2.0000	10.0000	6	39.4160	3
6.0000	2.0000	4.0000	6	593.5680	2
6.0000	2.0000	5.0000	4	74.3520	1
6.0000	2.0000	6.0000	6	596.2880	6
6.0000	2.0000	7.0000	4	74.1720	2
6.0000	2.0000	8.0000	6	110.3920	3
6.0000	2.0000	9.0000	6	110.3680	4
6.0000	2.0000	10.0000	6	77.1040	2
6.0000	5.0000	4.0000	6	595.0400	4
6.0000	5.0000	5.0000	4	1.7360	4
6.0000	5.0000	6.0000	6	31.9880	6
6.0000	5.0000	7.0000	6	151.4400	2

This table clearly shows that the vast majority in instances of non-integrated first phase percepts were B--- (pattern 6) and A- (pattern 4) in condition 2. This is entirely consistent with previous reports of first phase segregation (Deike et at 2013, Denham et al 2013). The 7 instances of conditions other than condition 2 with non-integrated first phase reports show no consistent pattern, and there are too few of them to draw firm conclusions.
